# Can GLP-1 Be a Target for Reward System Related Disorders? A Qualitative Synthesis and Systematic Review Analysis of Studies on Palatable Food, Drugs of Abuse, and Alcohol

**DOI:** 10.3389/fnbeh.2020.614884

**Published:** 2021-01-18

**Authors:** Candan Yasemin Eren-Yazicioglu, Arya Yigit, Ramazan Efe Dogruoz, Hale Yapici-Eser

**Affiliations:** ^1^Koç University, Research Center for Translational Medicine (KUTTAM), Istanbul, Turkey; ^2^School of Medicine, Koç University, Istanbul, Turkey; ^3^Department of Neuroscience, University of Chicago, Chicago, IL, United States; ^4^Department of Psychiatry, School of Medicine, Koç University, Istanbul, Turkey

**Keywords:** GLP-1, reward, food intake, mood, cocaine, amphetamine, alcohol, nicotine

## Abstract

The role of glucagon-like peptide 1 (GLP-1) in insulin-dependent signaling is well-known; GLP-1 enhances glucose-dependent insulin secretion and lowers blood glucose in diabetes. GLP-1 receptors (GLP-1R) are also widely expressed in the brain, and in addition to its role in neuroprotection, it affects reward pathways. This systematic review aimed to analyze the studies on GLP-1 and reward pathways and its currently identified mechanisms.

**Methods:** “Web of Science” and “Pubmed” were searched to identify relevant studies using GLP-1 as the keyword. Among the identified 26,539 studies, 30 clinical, and 71 preclinical studies were included. Data is presented by grouping rodent studies on palatable food intake, drugs of abuse, and studies on humans focusing on GLP-1 and reward systems.

**Results:** GLP-1Rs are located in reward-related areas, and GLP-1, its agonists, and DPP-IV inhibitors are effective in decreasing palatable food intake, along with reducing cocaine, amphetamine, alcohol, and nicotine use in animals. GLP-1 modulates dopamine levels and glutamatergic neurotransmission, which results in observed behavioral changes. In humans, GLP-1 alters palatable food intake and improves activity deficits in the insula, hypothalamus, and orbitofrontal cortex (OFC). GLP-1 reduces food cravings partially by decreasing activity to the anticipation of food in the left insula of obese patients with diabetes and may inhibit overeating by increasing activity to the consumption of food in the right OFC of obese and left insula of obese with diabetes.

**Conclusion:** Current preclinical studies support the view that GLP-1 can be a target for reward system related disorders. More translational research is needed to evaluate its efficacy on human reward system related disorders.

## Introduction

GLP-1 is an incretin hormone, derived from preproglucagon and released mostly by the L-cells of intestines (Lovshin and Drucker, 2009). Through its peripheric effects as inducing insulin secretion from pancreatic beta cells, gut emptying, and inhibiting glucagon secretion; its analogs are used in the treatment of type 2 diabetes mellitus (T2DM) (Zander et al., 2002). GLP-1 can cross the blood-brain barrier and access the nervous system. It can also be produced by neurons and microglial cells (Kappe et al., 2012). It acts through G-protein coupled GLP-1 receptors (GLP-1R), that act via the activation of adenylyl cyclase (Drucker et al., 1987) and protein kinase A, which induces gene transcription (Drucker, 2006). A recent study on detailed localization and characterization of GLP-1R in the brain reported that GLP-1Rs are highly expressed in mostly GABAergic neurons within the lateral septum (LS), hippocampus, bed nucleus of the stria terminalis (BNST), and amygdala (Graham et al., 2020).

GLP-1 analogs can decrease apoptosis, increase cell viability, neurogenesis, reduce inflammation, and decrease oxidative stress. GLP-1 reduces apoptosis by increasing levels of anti-apoptotic proteins as bcl-2 and decreasing levels of pro-apoptotic proteins as cytochrome c, caspase3, and bax. GLP-1 restores neuronal growth and increases cell viability by elevating cAMP, PKA, and CREB levels and altering the phosphorylation levels of GSK-3b, AKT, ERK, and mTOR, which results in other downstream changes important for cell survival. GLP-1 also promotes neurogenesis by stimulating neurotrophic factors as GDNF, VEGF, and BDNF and reduces inflammation by decreasing TNF-a, IL-6, IL-10, and microglial activation (Erbil et al., 2019).

In addition to its role in neuroprotection, current evidence showed that GLP-1 affects reward pathways. GLP-1Rs are widely expressed in areas of the mesolimbic reward pathway that receive direct projections from the nucleus tractus solitarius (NTS) (Alhadeff et al., 2012). GLP-1Rs are located as opposed to dopamine terminals in the caudal and rostral lateral septum (LS) (Reddy et al., 2016), and GLP-1 antagonist was shown to reduce lithium chloride-induced suppression of Nucleus accumbens (NAc) phasic dopamine release (Fortin et al., 2016). Furthermore, the expression of GLP-1R was highest in the LS compared to all other regions, and these GLP-1 neurons were colocalized with dopamine receptor and calbindin-expressing cells in the LS (Graham et al., 2020). A recent study on the effect of GLP-1 on dopamine activity showed that while GLP-1 increased DA uptake, DA clearance, and DAT surface expression in the striatum in rats (Jensen et al., 2020). Due to its effect on ventral tegmental area (VTA) and striatal dopamine levels, it is suggested that both peripheral and central GLP-1 regulates hunger, satiety, and body weight (Kenny, 2011). GLP-1R in the mesolimbic reward system specifically influences the control of hedonic eating (Hernandez and Schmidt, 2019). Moreover, in humans, exenatide, a GLP-1 receptor agonist, was shown to increase brain responses to palatable food consumption and decrease brain responses to the anticipation of palatable food consumption compared to placebo, along with significantly reduced food intake in obese patients with and without T2DM (van Bloemendaal et al., 2015a).

In addition to altering food satiety signals, GLP-1 is suggested to modulate “satiety” in drugs of abuse and alter reward-related changes in multiple drugs of abuse such as cocaine (Hernandez et al., 2018, 2019), alcohol (Egecioglu et al., 2013c), nicotine (Egecioglu et al., 2013a), and amphetamine (Egecioglu et al., 2013b).

Even though the role of GLP-1 on neuroprotection is well-known and new clinical trials have been started for its repurposing in neuroprotection, the role of GLP-1 on reward systems and its potential clinical use for reward modulation still need further studies. Therefore, in this systematic review, it was aimed to analyze the studies on GLP-1 and reward, establish a comprehensive framework on the reward-related effects of GLP-1, and answer its potential use for targeting reward system related pathologies.

## Methods

### Search Strategy and Study Selection

This study was conducted in line with the suggested PRISMA (Moher et al., 2009) and MOOSE (Stroup et al., 2000) guidelines. Web of Science and Pubmed were searched as the databases to reach relevant articles that include “glucagon-like peptide-1,” “GLP-1,” “glucagon-like peptide-1 receptor” or “GLP-1R” in their title, abstract, or as a keyword. “Reward” was not used as a keyword at this step. The search was done on September 5, 2018, and updated twice on December 27, 2019, and November 20, 2020. Duplicates between the two databases were removed in EndNote. Overall, 26,539 articles on Web of Science and Pubmed were identified. All articles were downloaded as abstracts. The methodological steps used were similar to our previous study (Erbil et al., 2019). Three authors synchronously probed the abstracts for identifying possible articles that included relevant information with a particular emphasis on GLP-1 and its effect on palatable food intake and addiction-related studies to cover reward system related disorders. Abstracts not about psychiatry, neurology, or neuroscience were excluded. In the identification step, original articles, in addition to reviews, were evaluated in full text to identify references that may have been missed by our search strategy. Reviews were screened for their references, and related citations not found by our initial search were included in the systematic review. In the screening step, two authors synchronously read all abstracts for inclusion. At this level, the main focus was on DPP-IV inhibitors, GLP-1R agonists, and antagonists. In case abstracts were inconclusive, the full article was accessed to study their relevance. Poster and conference proceedings and articles in other languages than English were excluded as quality criteria. Another author checked the final lists. Both preclinical and clinical studies were included to present a translational view and the current status of the research on this topic.

### Data Extraction

Studies were grouped for models of palatable food intake and drugs of abuse, considering different pharmacological pathways that they might be associated with. For preclinical studies, mouse/rat line, design of the study, GLP-1R agonist or DPP-IV inhibitor name, dose, administration method and duration, experimental groups in the study, molecular/behavioral/electrophysiological assessments, and main results were extracted. For human studies, the study sample size and design, GLP-1R agonist/antagonist or DPP-IV inhibitor name, dose, administration method and duration, assessments, and main results were extracted. Studies that focused on behavior but did not assess their relationship with GLP-1, such as behavioral or molecular change, were excluded. At least two authors have checked all steps.

### Quality Assessment and Evaluation of Findings

As a quality assessment, it was expected for articles to fully explain the design and usage of GLP-1, along with the design of molecular and behavioral outcome measures. Results were given as organized tables for each topic, and studies were given in chronological order in the tables. Studies that included data on more than one reward-related system as Sirohi et al. (2016), where the effect of GLP-1 agonists on palatable food intake, alcohol, and amphetamine was reported, and Suchankova et al. (2015), where the effect of GLP-1 on alcohol was assessed in both humans and mice, are presented in all related tables. Results are demonstrated first on the effects of GLP-1 on rewarding behaviors, secondly, its neuroanatomical explanations, and lastly, molecular mechanisms. A meta-analysis and publication bias analysis could not be conducted due to the heterogeneity of the study designs.

## Results

### Description of the Included Studies and the Study Characteristics

**Figure 1:**
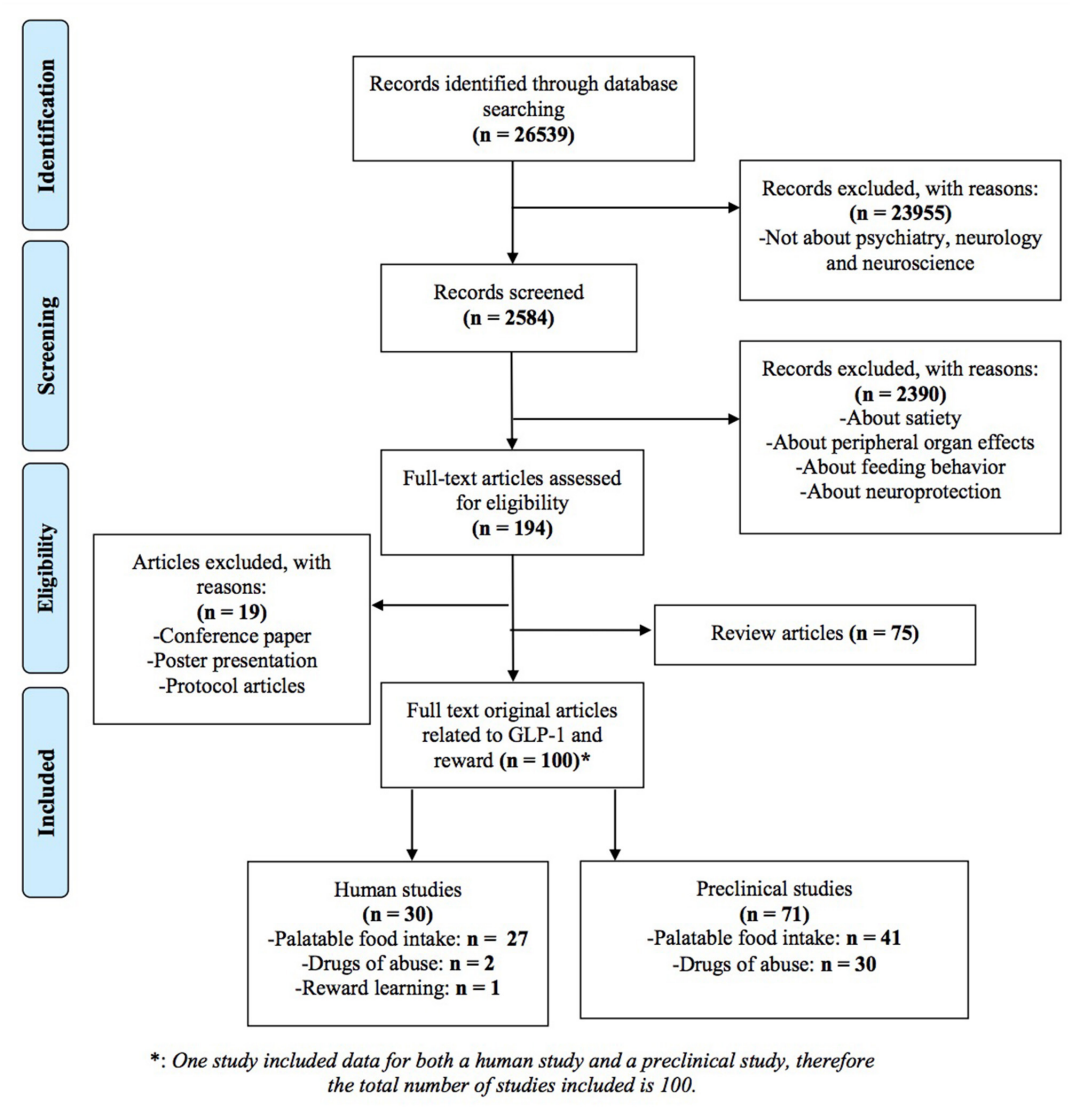
The PRISMA flow diagram of the search strategy.

**Figure 2:**
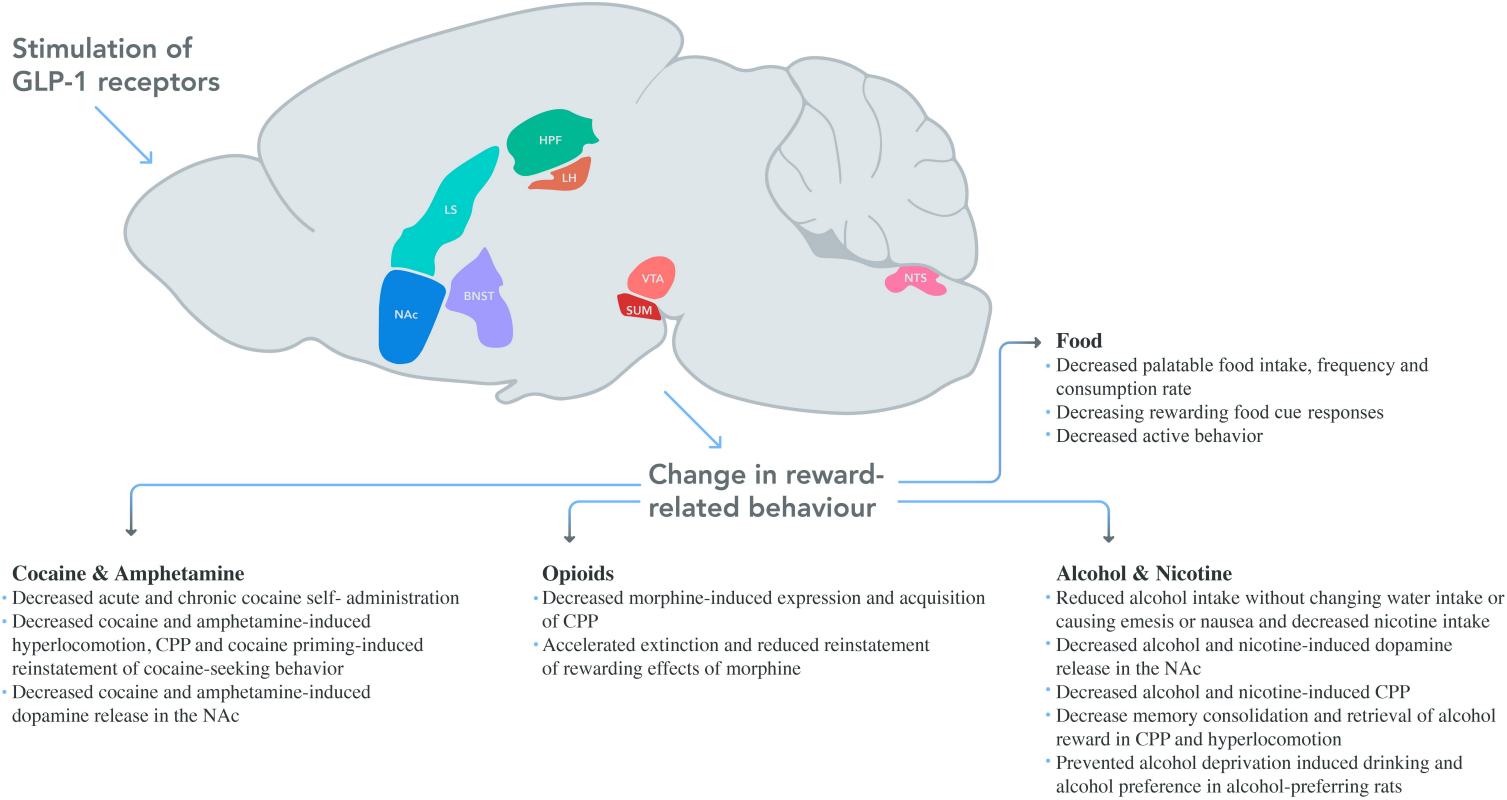
Schematic drawing of reversed reward responses after GLP-1 modulation. In addition to the systematic injection of GLP-1 agonists' effects on the brain; LH, VTA, NAc, NTS, SUM, and LS were studied for palatable food, whereas VTA, NAc, and NTE were studied for alcohol, NAc was studied for amphetamine, NAc and LS were for cocaine and NTS was for nicotine use behavior. Areas were drawn based on Allen Mouse Brain Atlas (NAc, nucleus accumbens; LS, lateral septum; HPF, hippocampal formation; LH, lateral hypothalamus; VTA, ventral tegmental area; SUM, supramammillary nucleus; NTS, nucleus tractus solitarius; BNST, bed nucleus of the stria terminalis).

After identification, screening, and data extraction, 100 studies were included for the systematic review. The PRISMA flow diagram of the search strategy can be found in Figure 1. Full details of the evaluated studies are given in Supplementary Tables, with a classification based on the rewarding target, rat or mice group, and chronological order. Summaries are presented for each rewarding category below. Neuroanatomical associations studies for each substance and schematic drawing of reversed reward responses after GLP-1 modulations are given in Figure 2.

### Preclinical Studies About Palatable Food Intake and GLP-1

All preclinical studies that assessed palatable food intake and its association with GLP-1 are given in Supplementary Table 1. In summary, evidence in rats and mice points out that GLP-1 analogs mainly decrease palatable food intake (Mathes et al., 2012; Hansen et al., 2014; Wright and Rodgers, 2014) in doses that do not affect blood glucose levels (Asarian et al., 1998; Yamaguchi et al., 2017; Maske et al., 2018; Gabery et al., 2020; Vestlund et al., 2020). Behavioral investigations of GLP-1 and palatable food intake also reported that injection of exendin-4 (Ex-4) (1 or 3.2 μg/kg IP doses only and not higher doses) also changed acquisition of food aversion learning (Liang et al., 2013) and that liraglutide (10 mg/kg IP) decreased rewarding cue responses in both chow and western diet (Jones et al., 2019). Below, neuroanatomical effects and molecular pathways associated with the GLP-1 control of palatable food intake are described.

### Neuroanatomical Pathways About GLP-1 and Palatable Food Intake

Many studies investigated the role of GLP-1R modulation in different neuroanatomical areas on palatable food intake (Supplementary Table 1). Here, each regional effect is summarized. The first study on the topic by Asarian et al. (1998) suggested that intracerebroventricular infusions of GLP-1 inhibit sham feeding by decreasing the orosensory positive feedback that drives licking. Vagal afferents damaged by surgery protected against Ex-4 mediated conditioned flavor avoidance, and it was suggested that PVN activation by GLP-1 might be dependent on vagal afferents (Labouesse et al., 2012).

Lateral hypothalamus (LH) is a core region for palatable food intake (López-Ferreras et al., 2018). Previous eating habits as chronic consumption of palatable foods, modeled with cafeteria diet in mice, changed GLP-1 mRNA expression in the hypothalamus and led to less responsiveness to Ex-4 in food reward behaviors (Mella et al., 2017). LH injections of GLP-1/estrogen reduced food intake more than GLP-1 or estrogen-only, which showed that this effect might be sex-biased (Vogel et al., 2016).

Moreover, NAc and VTA are the core cites for reward processing and are mostly studied for this topic (Dickson et al., 2012; Dossat et al., 2013). IP or VTA injection of Ex-4 decreased ghrelin-induced food intake (Howell et al., 2019). When Ex-4 is injected into VTA, it both decreased palatable food intake, 24 h of chow intake, and body weight; however, when injected into the core or shell of NAc, it did not affect normal chow but decreased high-fat food intake (Alhadeff et al., 2012). In mice, highly palatable high-fat food intake was reduced by the activation of GLP-1 neurons (Wang et al., 2015). When NAc core is studied, anorexia observed after NAc GLP-1 injections were suggested not to be due to viscerosensory stress or nausea but due to increased satiety. Intra-lateral ventricle (LV) GLP-1 injections of 1 and 3 μg intra-LV GLP-1 injections reduced food intake, whereas lower doses had no effect (Dossat et al., 2011). Intra-NAc core Ex-4 also successfully reduced μ-opioid receptor agonist (DAMGO) induced palatable food intake, and combination of intra-NAc core Ex-9 and DAMGO increased length of food intake (Pierce-Messick and Pratt, 2020). Viral modulation of GLP-1 expression on NTS (Alhadeff et al., 2017) or modulation of NTS by Ex-4 injection in rats (Alhadeff and Grill, 2014) or activation of NTS GLP-1 neurons in mice (Wang et al., 2015) decreased palatable food intake. When only chow was presented, intra-NTS Ex-4 also reduced chow intake along with bodyweight but did not change locomotor activity or induce a pica response, which is a model of nausea (Richard et al., 2015). Similar to LH, the effects on NTS was also found to be regulated by estrogen. NTS injections of the GLP-1/estrogen reduced food intake and caused a trend toward decreased bodyweight more than GLP-1 or estrogen alone (Vogel et al., 2016). NTS GLP-1 neurons project on the lateral parabrachial nucleus (LPBN), and Ex-4 injection of LPBN neurons showed similar effects on both chow and high-fat food intake (Alhadeff et al., 2014). Furthermore, semaglutide induced neuronal activation overlapped with meal-termination neuronal pathways controlled by PBN neurons (Gabery et al., 2020).

Expression of supramammillary nucleus (SuM) GLP-1R is comparable with the expression in the LH and NAc, but lower than that found in the NTS (López-Ferreras et al., 2019). Activation of GLP-1R by infusion of Ex-4 (0.01 and 0.03 μg) into the SuM or AAV virus in male or female rats was found to be potently anorexigenic. SuM GLP-1R controlled food reward predominantly in male rats and with a lower degree in females (López-Ferreras et al., 2018). Also, SUM injection of GLP-1/estrogen reduced food reward and food intake more than GLP-1 or estrogen alone (Vogel et al., 2016).

The intra-BNST GLP-1 decreased chow intake at a dose-dependent magnitude of effect, while exendin9-39 (Ex-9) reversed it. GLP-1 injection into the BNST less effectively suppressed feeding in high-fat diet (HFD) at 1 and 2 h (more significant at higher doses), and Ex-9 did not change intake in HFD mice (Williams et al., 2018).

The LS is a relay center for connections from the CA3 of the hippocampus to the VTA. Hippocampal formation (HPFv) Ex-4 decreased chow intake, meal size but did not affect meal frequency, and HPFv Ex-9 increased food intake only at the 6th hour. HPFv Ex-4 increased preference for the chow diet over the Western diet (Hsu et al., 2015). Ventral hippocampal field CA1 (vCA1) Ex-4 reduced food intake and operant responding for palatable food and this was modulated via vCA1 to medial PFC (mPFC) projections (Hsu et al., 2018). Intra-dLS GLP-1 decreased active lever presses; however, intra-LS Ex-4 with Ex-9 (at subthreshold doses) did not change chow intake, indicating GLP-1 in LS do not control *ad libitum* feeding. Ensure intake (large meal) before test sessions decreased subsequent chow intake, which was reversed with intra-LS Ex-9 (Terrill et al., 2019). In rats as well, intra-LS Ex-4 decreased overnight chow and HFD intake. The average dark-phase meal size and average light-phase meal size were decreased after intra-LS Ex-4 (Terrill et al., 2016).

The effect of GLP-1 on food intake can also be related to the circadian rhythm. Animal models show that blood GLP-1 levels can vary during the day based on mealtimes, and this variation may be independent of the insulin variation (Dailey et al., 2012). GLP-1 is not mainly dependent on Melanocortin-3 or 4 receptors (MC3R or MC4R), or any other postulated Agouti-related protein (AgRP) sensitive pathway for their function for food intake behavior (Edwards et al., 2000). Night/light eating changes also were defined by Alhadeff et al. (2017).

### Molecular Pathways About GLP-1 and Palatable Food Intake

Evidence suggests a significant role of GLP-1 in modulating *dopaminergic circuits* and molecular synthesis of dopamine. Mesolimbic tyrosine hydroxylase (TH) and dopamine receptor 1 (D1R) gene expression were significantly decreased in chronic HFD-fed rats; Ex-4 and food restriction reduced these decreased expressions (however, only D1R changes reached significance) (Yang et al., 2014). In mice, GLP-1/dexa reduced the expression of reward-related genes in the NAc such as D1R, dopamine receptor 2 long isoform (D2rlg), kappa opioid receptor (Kor), glucocorticoid receptor (Gr), others such as TH and dopamine transporter (DAT) were also decreased but non-significantly (Decarie-Spain et al., 2019). Intra-NTS Ex-4 in rats increased dopamine-B-hydroxylase expression in NTS, an enzyme for noradrenaline synthesis, and GLP-1 fibers and noradrenergic neurons were found to be colocalized in the NTS (Richard et al., 2015). In the VTA, intra-NTS changed TH (Mietlicki-Baase et al., 2013; Richard et al., 2015) and D2 expression levels (Richard et al., 2015). In a more detailed analysis of dopaminergic receptors, central Ex-4 injection (0.3 μg) increased the levels of dopamine metabolites, DOPAC and HVA, as well as dopamine turnover in the amygdala (Anderberg et al., 2014). Central Ex-4 decreased food intake at the 1st hour, without affecting food motivated operant behavior, by increasing amygdala dopamine, and it was not related to D2/D3 receptor blockage. In contrast, the 24-h chow-intake reduction produced by Ex-4 was significantly attenuated by the D2/D3 receptor blockade. Ex-4 significantly reduced operant behavior for a sucrose reward, and this reduction was not attenuated by the D2/D3 receptor blockade (Anderberg et al., 2014). Ex-4 also decreased sucrose induced licking behavior, and the magnitude of cue evoked phasic dopamine activity; this response of dopamine was significantly associated with subsequent sucrose directed behavior (Konanur et al., 2020).

Another neurotransmitter system GLP-1 modulates is the *glutamatergic neurotransmission*. In mice, Ex-4 application to TH+ VTA-to-NAc projecting DA neurons suppressed AMPA-R-mediated excitatory post-synaptic potentials (EPSCs) without changing NMDA EPSCs (Wang et al., 2015). In another study by Mietlicki-Baase et al. (2013) in rats, intra-VTA Ex-4 was found to reduce high-fat food intake (from 3 to 24 h), and these reductions were suppressed by AMPA/kainate receptor antagonist, CNQX. NMDA-R was not involved in food intake and the meal size suppressive effects of intra-VTA Ex-4. Ex-4 increased sEPSC frequency in VTA dopamine without changing sEPSC decay, time, peak amplitude, and charge transfer, which indicated a presynaptic GLP-1R activation on glutamatergic terminals in AMPA/kainate receptors. When these interactions were studied for NAc (Mietlicki-Baase et al., 2014), Ex-4 treatment did not alter dopamine release in NAc core slices. Ex-4 increased the frequency of NAc core medium spiny neuron (MSN) mEPSCs but did not affect kinetics or amplitude, indicating a presynaptic effect of GLP-1R activation. Ex-4 bath application decreased the paired-pulse ratio (PPR) of evoked EPSCs, further supporting a presynaptic effect by increasing the probability of glutamate release. Ex-4 suppressed action potential (AP) firing on PVT-to-NAc projecting neurons (Ong et al., 2017). Ex-4 induced suppression of AP firing was smaller and delayed when given with synaptic blockers (CNQX for glutamate receptors, PTX for GABA receptors). Ex-4 caused hyperpolarization of PVT-to-NAc neurons in the presence of synaptic blockers. However, in mice, GLP-1 reversibly depolarized or hyperpolarized BNST neurons, which was the opposite of when dopamine was applied, indicating excitatory and inhibitory responses in BNST (Williams et al., 2018).

In addition to other neuromodulators affected by GLP-1 agonists, Ex-4 increased pro-opiomelanocortin (POMC) expression while decreasing neuropeptide Y (NPY), and this effect was independent of the reduction of food intake and body weight (Yang et al., 2014). Estrogen receptors and GLP-1R are co-localized in areas involved in reward behavior regulation such as the VTA and NAc; central ERα signaling might be modulating the actions of GLP-1 on food-reward behavior (Richard et al., 2016).

### Preclinical Studies About Drugs of Abuse and GLP-1

Among the drugs of abuse that have been studied, cocaine (Egecioglu et al., 2013b; Graham et al., 2013; Harasta et al., 2015; Sørensen et al., 2015; Reddy et al., 2016; Schmidt et al., 2016; Sirohi et al., 2016; Fortin and Roitman, 2017; Hernandez et al., 2018, 2019; Bornebusch et al., 2019; You et al., 2019; Łupina et al., 2020), amphetamine (Lautar et al., 2005; Erreger et al., 2012; Egecioglu et al., 2013b; Sirohi et al., 2016), opioids (Bornebusch et al., 2019; Łupina et al., 2020; Zhang et al., 2020), alcohol (Egecioglu et al., 2013c; Shirazi et al., 2013; Sirohi et al., 2016; Sørensen et al., 2016; Vallöf et al., 2016, 2019a,b, 2020; Thomsen et al., 2017, 2019; Abtahi et al., 2018; Dixon et al., 2020), and nicotine (Egecioglu et al., 2013a; Tuesta et al., 2017) were found. All preclinical studies that assessed the association between GLP-1 and drugs of abuse are presented in Supplementary Table 2. Findings from each drug of abuse are summarized below.

#### Cocaine

Studies show a link between GLP-1 levels, GLP-1R modulation, and cocaine use (Supplementary Table 2). GLP-1 levels increased after cocaine use (You et al., 2019). Ex-4 significantly reduced cocaine-induced conditioned place preference (CPP) even at the lowest dose without affecting locomotor activity or causing aversion (Graham et al., 2013). Ex-4 decreased acute and chronic cocaine self-administration and D1R agonist-induced hyperlocomotion along with cocaine-induced c-fos expression and dopamine release in the striatum in mice (Sørensen et al., 2015). Ex-4 decreased cocaine-induced hyperlocomotion and CPP along with accumbal dopamine release without affecting spontaneous locomotor activity and accumbal dopamine release in normal conditions (Egecioglu et al., 2013b; Sørensen et al., 2015). On the first day of extinction, GLP-1 levels were increased to the same level seen in the cocaine self-administration period; however, it was normalized on the 14th day of extinction (You et al., 2019). Low doses of peripheral Ex-4 (0.1 and 0.2 μg/kg) reduced cocaine priming-induced reinstatement of cocaine-seeking behavior dose-dependently but did not affect chow food intake, meal patterns, and body weight (Hernandez et al., 2018, 2019). When intra-VTA was injected, Ex-9 inhibited the effects of peripheral Ex-4 (Hernandez et al., 2018), and VTA GLP-1R knockdown (KD) significantly increased cocaine intake (Schmidt et al., 2016). Moreover, intra-VTA Ex-4 (0.05 μg/kg) also reduced cocaine-seeking dose-dependently but did not affect sucrose seeking (Schmidt et al., 2016; Hernandez et al., 2018). Extinction following cocaine exposure diminished preproglucagon (PPG) expressions in the NTS (Hernandez et al., 2018). Ex-4 reduced cocaine-induced increase in dopamine concentration in the NAc core but not the shell, without affecting the dopamine reuptake (Fortin and Roitman, 2017). Cocaine priming-induced reinstatement of cocaine-seeking behavior was attenuated in rats Ex-4 administered into the NAc core (0.005 and 0.05 μg/500 nl) and shell (only the higher dose) without again changing sucrose seeking. Ex-4 increased MSN AP frequency in the NAc core and shell following the extinction of cocaine self-administration without changing sEPSC frequency, paired-pulse ratio (PPR), or sEPSC. These results were not associated with GLP-1R expression levels in the NAc after voluntary cocaine intake (Hernandez et al., 2019).

Cocaine-experienced rats had greater plasma corticosterone levels, and corticosterone administration into the hindbrain fourth ventricle reduced cocaine self-administration dose-dependently without changing sucrose intake, and these effects were inhibited by GLP-1 antagonist (Schmidt et al., 2016).

In the LS slices, GLP-1 increased DAT surface expression (increased transport capacity), and DA uptake and GLP-1 antagonist reversed these effects. Systematic administration of Ex-4 of 2.4 μg/kg decreased LS activity and cocaine-induced extracellular DA release in the LS along with reduced expression of retrograde messenger 2-AG and arachidonic acid (AA), which serves as a reducing agent of DAT function (Reddy et al., 2016). Also, GLP-1R mRNA expression was high in the GABAergic neurons of the dorsal lateral septum (dLS). In the dLS, GLP-1R deficient mice neurons were shown to be more excitable. GLP-1R deficient mice had increased cocaine-induced CPP and cocaine-induced locomotor activity. GLP-1R gene delivery to the dLS of GLP-1R deficient mice resulted in reduced cocaine-induced CPP and locomotor activity without changing anxiety behaviors (Harasta et al., 2015).

#### Amphetamine

Ex-4 (2.4 μg/kg) decreased amphetamine-induced hyperlocomotion, CPP, and accumbal dopamine release without affecting spontaneous locomotor activity and accumbal dopamine release in normal conditions (Egecioglu et al., 2013b). It reduced basal and amphetamine-induced locomotor activity in rats in very high doses (30 μg/kg). Therefore, GLP-1 agonists were suggested as potential targets for psychostimulant abuse (Erreger et al., 2012). This finding was also supported by the use of DPP-IV inhibitor, AMAC, which dose-dependently decreased amphetamine-induced hyperactivity (Lautar et al., 2005). In GLP-1R KD Nestin mice, Ex-4 blockage of amphetamine-induced CPP was not observed (Sirohi et al., 2016).

#### Opioids

Linagliptin, a DPP-IV inhibitor, inhibited the expression and acquisition of morphine-induced CPP along with accelerating the extinction and reducing reinstatement of the rewarding effects of morphine (but only at the lower dose), without affecting the locomotor activity in rats (Łupina et al., 2020). Contrary to this finding, in mice, Ex-4 did not impact morphine-induced CPP, morphine withdrawal, or hyperlocomotion. Ex-4 also did not reduce remifentanil (a synthetic opioid) self-administration (Bornebusch et al., 2019). However, both systematic and intra-NAc shell Ex-4 successfully decreased oxycodone self-administration and cue priming-induced reinstatement of oxycodone seeking-behavior in mice without causing adverse feeding behaviors or changing analgesic effects of oxycodone (Zhang et al., 2020).

#### Alcohol

In rats, Ex-4 successfully reduced ethanol intake dose-dependently and reduced spontaneous locomotion (Bornebusch et al., 2019). Ex-4 reduced alcohol intake and alcohol-seeking behavior in rats after 8 months of alcohol use (Egecioglu et al., 2013c). Ex-4, at doses not affecting baseline locomotor activity, reduced alcohol-induced locomotor behavior, and accumbal dopamine release evoked by ethanol. Ex-4 (3.2 μg/kg) decreased intravenous ethanol self-administration but did not change palatable liquid food intake (Sørensen et al., 2016). In alcohol dependent mice, an exenatide analog, AC3174, significantly reduced voluntary ethanol intake (Suchankova et al., 2015). Liraglutide and exenatide (to a lesser extent) reduced alcohol intake without affecting water intake or causing emesis or nausea in monkeys (Thomsen et al., 2017, 2019). Acute liraglutide attenuated the alcohol-induced increase in accumbal dopamine, decreased alcohol-induced CPP, and prevented alcohol deprivation-induced drinking. After 12 weeks of alcohol consumption, both acute and chronic liraglutide administration reduced alcohol intake (along with food intake). In alcohol-preferring rats, chronic liraglutide decreased operant alcohol self-administration (Vallöf et al., 2016). Long-term treatment (9 or 5 weeks) of dulaglutide reduced ethanol intake and ethanol preference in both male and female rats and altered levels of dopamine, serotonin, and noradrenalin in the amygdala of male rats and dopamine, DOPAC, and noradrenaline levels in the striatum of female rats (Vallöf et al., 2020).

When neuroanatomical associations of this behavioral effect were analyzed, intra-NTS Ex-4 (0.05 μg per side) decreased alcohol-induced locomotor behavior, accumbal dopamine release, and memory consolidation of alcohol reward in mice, whereas a lower dose (0.025 μg per side) was ineffective. Ex-9 injected into the NTS blocked the reducing effect of systematic Ex-4 on locomotor activity in mice (Vallöf et al., 2019b). In another study, both peripheral GLP-1 and Ex-4 decreased alcohol intake, and GLP-1 reduced alcohol preference in the CPP test in rats, whereas Ex-9 increased alcohol intake (Shirazi et al., 2013). Ex-4 alone, or in combination with the ghrelin antagonist, in the NAc shell but not core, reduced alcohol intake in a time-dependent manner (Abtahi et al., 2018). Ex-4 into the NAc shell blocked alcohol-induced locomotor behavior, memory retrieval of alcohol reward in CPP, and decreased alcohol intake without affecting water intake and body weight. GLP-1R expression in the NAc shell (but not in the PFC, VTA, amygdala, hippocampus, and striatum) was also increased in high alcohol-consuming mice. Ex-4 into the posterior VTA reduced alcohol-induced locomotor behavior but did not change CPP or alcohol intake, and Ex-4 into the anterior VTA did not alter locomotor activity or CPP. Ex-4 into the laterodorsal tegmental area blocked alcohol-induced locomotor behavior and reduced alcohol intake but not CPP (Vallöf et al., 2019a). Intra-VTA Ex-4 significantly decreased alcohol self-administration without affecting food intake or locomotor activity, and this was more prominent in alcohol-preferring rats. However, intra-VTA Ex-4 did not change extinction following the reacquisition of alcohol self-administration or motivation for alcohol (Dixon et al., 2020).

#### Nicotine

Ex-4, at doses not affecting baseline locomotor activity, reduced nicotine-induced locomotor behavior, accumbal dopamine release, and blocked nicotine-induced CPP in mice. Ex-4 also abolished nicotine-induced locomotor sensitization (Egecioglu et al., 2013a). Also, Ex-4 (10 μg/kg) and sitagliptin decreased nicotine intake, whereas GLP-1R KO mice increased nicotine intake, but neither changed food responses. Chemogenetic activation of NTS GLP-1 neurons also decreased nicotine intake. NTS GLP-1 neurons activated medial habenular (MHb) projections to the interpeduncular nucleus (IPN), and GLP-1 application into the IPN decreased nicotine intake and attenuated nicotine reward without causing malaise; Ex-9 into the IPN reversed these effects. Neither changed food intake nor food reward (Tuesta et al., 2017).

### Human Studies About Palatable Food Intake, Cocaine, and GLP-1

All human studies of palatable food intake and GLP-1 are summarized in Supplementary Table 3. Only two studies assessed drugs of abuse and GLP-1 in humans. The one study on cocaine showed that GLP-1 levels were significantly reduced after cocaine injections in cocaine users, which might be precipitating further cocaine intake, and subjective “anxiety” was a positive predictor of post-cocaine GLP-1 concentrations (Bouhlal et al., 2017). Another study on alcohol use disorder (AUD) found that 168Ser allele (rs6923761) was associated with AUD in humans. Furthermore, 168Ser allele was associated with increased neuronal activity in the globus pallidus, an area crucial for reward processing, for high monetary reward during an incentive delay task (Suchankova et al., 2015). One study evaluated GLP-1s effect on binge episodes in T2DM patients with a history of binge eating disorder (Da Porto et al., 2020).

As indirect evidence about palatable food intake and GLP-1, blood levels of GLP-1 were analyzed in relation to food intake. GLP-1 levels did not correlate with the food wanting task scores after a 4-course meal either in staggered or non-staggered intake models. However, staggered meal intake resulted in higher GLP-1 levels and satiety, along with lower ghrelin and desire for food scores than non-staggered meal intake (Lemmens et al., 2011). Higher GLP-1 plasma levels before *ad libitum* food intake were associated with lower intake of carbohydrates and simple sugar but not total food intake (Basolo et al., 2019). When exposed to palatable food compared to non-palatable food, GLP-1 levels did not change significantly in obese patients (Rigamonti et al., 2015). Supporting this finding, acute exenatide decreased sodium and total food intake without affecting salt craving scores in healthy, obese, and T2DM obese groups. Prolonged liraglutide did not change sodium or total food intake along with salt craving scores (Smits et al., 2019).

Exenatide increased cerebral glucose metabolic rate (CMRglu) in regions related to glucose homeostasis regulation (frontal, occipital, temporal, parietal lobes, limbic system, insula, and putamen) and food reward (orbitofrontal lobe, thalamus, anterior, and posterior cingulate). It decreased CMRglu in the hypothalamus, along with reduced plasma insulin levels (Daniele et al., 2015). In a small sample of male patients, resting-state data also showed that co-administration of intragastric glucose and GLP-1 antagonist resulted in higher resting state functional connectivity (rsFC) between the hypothalamus and the left lateral orbitofrontal cortex (OFC), between the right NAc and the right lateral OF, and lower rsFC between the midbrain and the right caudate nucleus. Glucose ingestion only decreased prospective food consumption and increased sensations of fullness compared to baseline. These changes were not observed after co-administration with GLP-1 antagonists (Meyer-Gerspach et al., 2018). In a small sample of female patients, exenatide increased functional connectivity between left NTS and left hypothalamus and thalamus in obese and between right NTS and left thalamus in both obese and lean individuals. Exenatide also induced a positive correlation between hunger ratings and NTS functional connectivity in both obese and lean, with more significance in the obese (Coveleskie et al., 2017). In Roux-en-Y gastric bypass (RYGB) surgery patients, octreotide (a somatostatin analog) suppressed post-prandial plasma GLP-1 levels, which correlated with palatable food reward in a progressive ratio task, food appeal scores, and increased activation of important reward system areas such as NAc, caudate, anterior insula, and amygdala. These results were not seen in gastric banding (BAND) surgery patients or controls (Goldstone et al., 2016). Furthermore, Ex-9 resulted in decreased connectivity in the right middle frontal gyrus and right caudate nucleus pre-RYGB surgery and reduced connectivity in the right OFC post-RYGB surgery (van Duinkerken et al., 2020). In addition, Ex-9 more significantly increased the connectivity in the left lateral occipital cortex post-RYGB compared to pre-RYGB, and this was associated with a greater decrease in BMI, savory food appetite scores, and hunger scores (van Duinkerken et al., 2020).

Plasma GLP-1 levels showed a positive correlation with regional cerebral blood flow (rCBF) in the left dorsolateral prefrontal cortex (dlPFC) and hypothalamus independent of sex, age, adiposity, insulin, glucose, and free fatty acids (Pannacciulli et al., 2007). In a long-term lifestyle intervention, when GLP-1 and dlPFC activity co-occurred, it successfully predicted subsequent weight loss. However, neither GLP-1 nor dlPFC predicted weight loss individually (Maurer et al., 2019). Post-prandial GLP-1 levels increased from pre to post-RYGB and SG surgeries. In RYGB, this increase in GLP-1 correlated with decreased activity in the inferior temporal gyrus and right middle occipital gyrus and increased activity in the right medial PF gyrus/paracingulate cortex to high-energy vs. low-energy visual and auditory food cues (Baboumian et al., 2019).

Combined infusion of GLP-1 and PYY in fasted subjects reduced energy intake and decreased brain activity in the amygdala, caudate, insula, NAc, OFC, and putamen to food vs. non-food cues (De Silva et al., 2011). In lean controls, higher sugar intake was associated with reduced GLP-1 response to glucose intake. These responses showed negative correlations with dorsal striatum reactivity to food cues without having any correlations with NAc activity (Dorton et al., 2018). Colonic propionate injection, a short-chain fatty acid which acutely increased plasma GLP-1 and PYY levels, reduced brain activity in the caudate and NAc to high-energy food pictures in healthy lean men. Even though these changes were associated with decreased high-energy food appeal, they were independent from changes of GLP-1 or PYY levels (Byrne et al., 2016).

Independent of other metabolic and hormonal factors, exenatide decreased activation of the bilateral insula, left putamen, and right OFC to both food and high-calorie food vs. non-food pictures in T2DM patients and of the right insula and left OFC to high-calorie vs. non-food pictures in obese patients along with reduced food intake (van Bloemendaal et al., 2014). Exenatide decreased brain activation to the anticipation of palatable food in bilateral OFC of lean subjects, bilateral putamen, left insula, and left amygdala of the obese with T2DM, which might reduce food cravings. Exenatide increased brain activation to the consumption of palatable food in the right caudate nucleus of lean subjects, in the right OFC of obese subjects and left insula, bilateral putamen, and left amygdala of obese with T2DM, which might inhibit overeating. Ex-9 reversed these effects (van Bloemendaal et al., 2015a). Emotional eating was negatively correlated with exenatide-induced reductions of activation in the amygdala of obese and in the insula of T2DM patients, which showed that effects of GLP-1 were less significant on patients with emotional eating (van Bloemendaal et al., 2015b). The increase in GLP-1 levels after an oral glucose load was negatively correlated with food cue-induced OFC activity in both lean and obese individuals, and this was independent of changes in insulin, glucose concentrations, gender, BMI, and age. Only lean individuals showed associations between post-prandial insulin and OFC activations (Heni et al., 2015).

T2DM patients showed increased activity in the bilateral insula, left amygdala, right OFC in response to high-energy food cues during the fasting condition. Meal intake decreased bilateral insula activation, and GLP-1R antagonist reversed the meal-induced reductions in the bilateral insula activity in response to high-energy food, in addition to leading to increased hunger scores (Jennifer et al., 2015). In obese T2DM patients, GLP-1R agonist, liraglutide, decreased activation in the bilateral insula after fasting and left putamen after the post-prandial condition in the fMRI task while viewing both food and high-calorie food pictures at 10 days after treatment. This effect was not observed after 12 weeks of treatment (Jennifer et al., 2016). T2DM patients showed reduced activation in the right insula in response to palatable food compared to lean controls, and liraglutide increased the activation in the right insula and caudate nucleus at 10 days after treatment, but these effects were not observed after 12 weeks. Liraglutide decreased brain activity in the parietal cortex, insula, and putamen of the T2DM patients. While on liraglutide, the hunger and appetite scores of T2DM patients were positively correlated with activations in the precuneus, cuneus, parietal cortex, and nausea scores negatively correlated with activations in the cuneus, precuneus, cingulate cortex, and some parts of the PFC in response to highly desirable vs. less desirable food cues (Farr et al., 2016a). Short-term liraglutide administration in T2DM patients increased GLP-1, gastrointestinal peptide (GIP), and decreased percent change of leptin levels (Farr et al., 2016b). Liraglutide, when administered for a more extended period (5 weeks) at the highest dose, increased OFC activation to food vs. non-food cues when corrected for BMI/weight in obese patients (Farr et al., 2019). These results further support ten Kulve (Jennifer et al., 2016) as longer-term treatments of GLP-1 failed to reach significance in the weight loss process (ten Kulve et al., 2016). When compared to pre-surgery, GLP-1R antagonist administration more significantly increased activation in the caudate nucleus to visual food cues and in the insula to gustatory food cues after RYGB, indicating an effect of surgery on the actions of GLP-1R blockage (Jennifer et al., 2017).

## Discussion

To our knowledge, this study is the most up to date and comprehensive translational review of the effect of GLP-1 on reward. Our current review demonstrates that GLP-1 not only decreases palatable food intake, but it can also decrease cocaine, amphetamine, alcohol, and nicotine use in animal models (Supplementary Tables 1, 2). A limited number of human studies also support the central regulatory role of GLP-1 on reward pathway functional connectivity (Supplementary Table 3).

This review summarizes that the effect of GLP-1 is not just through its peripheric effects, but also through its central effects on reward processing as assessed by injection of GLP-1 analogs or GLP-1 itself to intra-NTS, NAc, VTA, LH, lPBN, SUM, BNST, LS for palatable food intake, intra-VTA, NAc, and LS for cocaine, intra-NAc for amphetamine, intra-NAc, NTS, dLS for alcohol, and intra-NTS and IPN for cocaine. It is shown to be increasing dopamine-B-hydroxylase levels, decreasing extracellular dopamine release, increasing DAT surface expression, increasing dopamine turnover, and suppressing projections to NAc, GLP-1R agonists might be decreasing palatable food or drug-induced phasic dopaminergic signaling in reward-related areas and therefore decreasing conditioned consummatory behavior.

As an example pathway to decrease conditioned consummatory behavior, VTA GLP-1R signaling potentially controls for palatable food intake by modulating the rewarding value of the ongoing meal while having fewer effects on between-meal satiety processes. Focusing on the molecular systems behind the dopaminergic modulation, it was shown that GLP-1R signaling in the VTA alters presynaptic modulation of glutamatergic excitation of dopamine neurons via AMPA/kainate but not NMDA receptors. AMPA/kainate but not NMDA receptors also mediate the effects of intra-NAc GLP-1R activation. On the opposite, in mice, GLP-1 reversibly depolarized or hyperpolarized BNST neurons, which was the opposite of when dopamine was applied, which indicated excitatory and inhibitory responses in BNST (Williams et al., 2018; Supplementary Tables 1, 2). When these effects were studied using cocaine, Ex-4 increased MSN action potential (AP) frequency in the NAc core and shell following the extinction of cocaine self-administration without changing sEPSC frequency or PPR or sEPSC, which indicated that increased excitability of MSNs in NAc might be contributing to suppressive effects of Ex-4 and this is a mechanism independent of the presynaptic stimulation (Hernandez et al., 2019). As a gap in the literature, many of the preclinical studies on drugs of abuse were based on behavioral data and the modulation of GLP-1 in only specific brain regions; further studies are needed that evaluate the molecular and electrophysiological background of these relationships, and that discriminate between the molecular effects of different substances.

As another significant finding of this study, current literature points that while GLP-1 treatment significantly decreases addictive-like behavioral effects on cocaine, alcohol, and nicotine, the evidence for opioid addiction is contradictory. One study reported that Ex-4 fails to reduce morphine-induced CPP, morphine withdrawal, or hyperlocomotion in mice, which indicates that GLP-1 may not be beneficial in the treatment of opioid addiction as it is in other drugs of abuse (Bornebusch et al., 2019). However, a recent study found that Ex-4 successfully decreases oxycodone self-administration and oxycodone-seeking behavior in mice without changing food intake or causing aversive behaviors (Zhang et al., 2020). These results are also replicated when Ex-4 is directly injected into the NAc shell (Zhang et al., 2020). This new data on opioids can serve as an indicator of using GLP-1 to reduce opioid reinforcement and drug relapse. The differences between studies can be due to species, strain, type of assays, or dose range; new and more standardized studies on opioids are necessary to better understand this relationship.

Some new studies also suggest the role of GLP-1 in the control of sexual behaviors. Both systematic and intra-NTS Ex-4 results in decreased sexual interaction behaviors in sexually naïve male mice (Vestlund and Jerlhag, 2020b). Furthermore, investigating specific regions important in reward neurocircuitry, Ex-4 injection into posterior VTA or NAc shell reduces pre-sexual interaction behaviors, and intra-LDTg Ex-4 reduces all phases of sexual interaction behaviors (Vestlund and Jerlhag, 2020a). Ex-4 into anterior-VTA does not result in significant changes in sexual behavior. These results support the role of GLP-1 in social rewarding behaviors and further highlight its effect on reward-related regions.

In terms of human studies, they have the disadvantage of having small sample sizes. Also, they focus mainly on the palatable food intake, except for one on cocaine. They lack evaluation of the mediators as chronic stress and intellectual level on the outcome. Here, the translation of preclinical studies to human studies is limited. When clinical trial registries are also investigated, it can be seen that upcoming studies also focus on diabetes or obesity. Studies are still limited on different drugs of abuse, alcohol, or nicotine.

As a summary of human studies, GLP-1 analogs modulated palatable food intake in humans. In T2DM patients, GLP-1 analogs modulated palatable food intake in humans. In T2DM patients, the insula has increased activity during fasting and less activity in response to palatable foods, while GLP-1R agonists reversed these activity differences. GLP-1 and dlPFC connectivity coupling also play a role in regulating palatable food intake (Supplementary Table 3). However, current evidence may indicate a role of GLP-1 in the induction of weight loss without affecting the long-term weight loss process. Plasma GLP-1 levels may correlate more with carbohydrate intake rather than total food wanting. However, in obese patients, the expected fluctuation of GLP-1 levels in response to food consumption may be lost, and weight control surgeries may restore these levels. When exposed to palatable food compared to non-palatable food, GLP-1 levels were not changed significantly in obese patients. These findings indicate that palatable food intake might be partially promoted due to insufficient GLP-1 response (Rigamonti et al., 2015; Supplementary Table 3). GLP-1 may change the functional connectivity of the hypothalamus and alter eating. As seen in animals, GLP-1R agonists reduce food cravings and increased brain activation to the consumption of palatable food in the right OFC of obese subjects and the left insula, bilateral putamen, left amygdala of obese patients with T2DM.

In our systematic review, we have observed that different substances such as palatable food or drugs of abuse have different effects on specific brain regions based on the dose, administration route, timing, satiety, along with other environmental factors. Hunger ratings were not associated with either GLP-1 or dorsal striatum activity. As a result of increased simple sugar intake, reduced GLP-1 mediated satiety signaling might result in sustained salience of food cues via dorsal striatum activity even after food intake and contribute to overeating and weight gain. In lean individuals, abnormal activities were not observed in the NAc as it did in the obese; therefore, hunger ratings might not be associated with GLP-1 levels in lean individuals.

The current literature on GLP-1 and reward has several limitations. Preclinical studies explored the short-term effects of GLP-1 analogs; however, knowledge about their long-term benefits is limited and needs to be investigated. A few clinical studies by Jennifer et al. (2016) and ten Kulve et al. (2016) found that longer-term GLP-1R agonist treatments may not be useful in the weight loss process as the changes seen after short-term treatment such as improvement of activity deficits in the insula and caudate nucleus in response to palatable food did not persist during the long-term treatment (12 weeks). Further studies with longer assessment periods are needed for both preclinical and clinical studies because of these contrasting findings. As another limitation, different GLP-1 analogs could not be distinguished for their effect on reward pathways since most of the studies used either Ex-4, liraglutide, linagliptin, or Ex-9 as an antagonist and did not compare them to each other. Previous literature on GLP-1 and neuroprotection (Erbil et al., 2019) showed that different GLP-1 analogs might diverge on their neuroprotective effect, and it was mostly time and dose-dependent as higher doses showed greater reversal of neurodegenerative processes and dual combinations were more effective in general. This effect might also be observed in the regulation of reward pathways, which needs to be further assessed.

This systematic review brings two possible future perspectives. Firstly, amphetamine administration results in hyperlocomotion, risk-taking behavior, and impaired cognition in mice resembling mania symptoms in bipolar disorder. The effect of GLP-1R agonists on reversing amphetamine-induced locomotion and that liraglutide may act similar to lithium in terms of reversing cognitive deficits and liraglutide decreased lithium-induced weight gain brought the idea that GLP-1 analogs can serve as a potential target for psychostimulant abuse and treatment for bipolar disorder since it can reverse several aspects of mania and drug-induced side effects in bipolar disorder (Chaves Filho et al., 2020). Along the same line, Ex-4 decreases amphetamine-induced locomotion without changing anxiety or aversive behaviors in rats (Erreger et al., 2012) along with reducing amphetamine-induced CPP and accumbal dopamine release (Egecioglu et al., 2013b). As limitations, both studies mentioned above use relatively high doses of GLP-1R agonist (30 and 2.4 μg/kg, respectively) and thus need further research.

Secondly, GLP-1 could have a significant role in psychological stress responses, in addition to mood disorders. GLP-1 could be a mediating molecule between mood, feeding, and reward-related behaviors. It has been shown by the cited studies above that GLP-1R exists on reward pathways, in addition to the amygdala, dorsal raphe, LS, hippocampus, and also BNST. These pathways also regulate mood and stress responses. A study showed that central GLP-1 administration increases anxiety-like behavior (Möller et al., 2002). GLP-1R KD of BNST neurons, specifically in rats, resulted in decreased anxiety-like behavior and stress-induced hypophagia (Zheng et al., 2019). Plasma corticosterone levels of GLP-1R KD were also increased in response to acute stress, while baseline corticosterone levels were similar between GLP-1R KD and control, indicating that actions of GLP-1 may be partially by limiting acute stress-induced plasma corticosterone production. In humans, one study reported that GLP-1R gene expression in the dlPFC was significantly different between healthy controls and mood disorder patients after controlling for age, sex, ethnicity, and BMI (Mansur et al., 2019). Emotional eating is negatively correlated with exenatide-induced reductions of activation in the amygdala of obese patients and in the insula of T2DM patients, which indicates an attenuated effect of GLP-1 on patients who suffer from emotional eating (van Bloemendaal et al., 2015b). Another study on GLP-1 polymorphisms found that two GLP-1 polymorphisms, A allele in rs10305492 and C allele in rs1042044, show associations with response bias in a probabilistic reward task (PRT), a laboratory-based measurement of anhedonia (Yapici-Eser et al., 2020). Based on these studies, future studies (both preclinical and clinical) should also assess mood as a construct of reward pathway associated with GLP-1.

In conclusion, GLP-1Rs are located in areas important for reward, and they can significantly alter palatable food or drug-induced dopamine levels and glutamatergic neurotransmission, which results in decreased palatable food intake and substance use. Preclinical evidence is accumulating, and translational research is highly required to increase knowledge on the effect of GLP-1 on reward systems.

## Data Availability Statement

The original contributions presented in the study are included in the article/Supplementary Material, further inquiries can be directed to the corresponding author/s.

## Author Contributions

HY-E and CYE-Y designed the study, wrote the protocol, and wrote the first draft of the manuscript. CYE-Y, AY, and RD managed the literature searches and analyses. All authors contributed to and have approved the final manuscript.

## Conflict of Interest

The authors declare that the research was conducted in the absence of any commercial or financial relationships that could be construed as a potential conflict of interest.
