# Supplementary material for: Can GLP-1 Be a Target for Reward System Related Disorders? A Qualitative Synthesis and Systematic Review Analysis of Studies on Palatable Food, Drugs of Abuse, and Alcohol
Source: Front Behav Neurosci. 2021 Jan 18;14:614884. doi: 10.3389/fnbeh.2020.614884 (PMC7848227; doi:10.3389/fnbeh.2020.614884)
Supplement: Supplementary file 1 [file Table_1.DOCX]

| Supplementary Table 1. Preclinical studies on GLP-1 and palatable food intake | | | | | |
| --- | --- | --- | --- | --- | --- |
| ***Name of First Author/ Publication Year*** | ***Mouse/ Rat Line & Age or weight*** | ***Definition of the Model/Application of GLP-1 agonist (dose and method)*** | ***Experimental Groups*** | ***Assessments*** | ***Main Findings*** |
| **1. Preclinical** | | | | | |
| **1.a Rats** | | | | | |
| (Asarian et al., 1998) | Male Sprague–Dawley rats | Rats received sham feeding for 2 weeks to stabilize 45 min of sham intake/GLP-1 (3, 10, or 30 μg) i.c.v. | 1. aCSF 2. GLP-1 (3 μg) 3. GLP-1 (10 μg) | Feeding, grooming, exploratory behavior consisting of sniffing, locomotion, rearing, resting, and anomalous behaviors 5 min after injection Sucrose sham-fed intakes during 0, 15, 30, 45 min intervals 5 min after injection Sucrose intake (numbers of sucrose licks, bursts, clusters, and interlick intervals) at 15 min start of each test | Sham feeding of 0.8 mol/L sucrose was inhibited (almost %50) by each GLP-1 doses at all times, but GLP-1 did not terminate sham feeding The frequency of sham feeding behavior (initial rate of licking, licks/minute) was reduced by GLP-1(3 and 10 μg) 3 μg of GLP-1 decreased feeding during 45-min of behavioral tests GLP-1 did not alter other behavioral categories measured The frequency of resting behavior did not change by GLP-1  GLP-1 (3 and 10 μg) reduced significantly burst size and cluster size |
| (Edwards et al., 2000) | Adult male Wistar rats (250–300 g) | ICV injected GLP-1 (3 nmol) or Agrp (83–132) in combination with GLP-1 | 1. ICV injected saline or a-melanocyte-stimulating hormone (a-MSH) at doses of 0.3, 1, 3 or 10 nmol 2. ICV injected saline or a-MSH at a dose of 1 nmol 3. ICV injected saline or CART 0.2 nmol, Agrp 1 nmol or CART and Agrp  4. ICV injected saline or GLP-1 3 nmol, CRF 0.3 nmol, Agrp 1 nmol, Agrp in combination with GLP-1 | Food intake was measured at 1, 2, 4, 8, and 24 h | 1 nmol a-MSH significantly reduced food intake at 1 h and 2 h and 1 nmol Agrp inhibited this anorectic effect  CART reduced 1, but not 2 h food intake and Agrp did not affect this result GLP-1 significantly reduced 1, 2, 4, and 8 h food intake, CRF significantly reduced 1, 2 and 4 h food intake and Agrp did not affect these results at each time point CART, GLP-1, and CRF, unlike leptin, were not mainly dependent on MC4-R or MC3-R or any other postulated Agrp sensitive pathway for their function |
| (Dossat et al., 2011) | Naive male Wistar rats (325 g) | Intra-Lateral ventricle (LV) 0, 0.033, 0.15, 0.3, 1, or 3g of GLP-1 Intra-NAc core 0.1, 0.025 μg of GLP-1/ 0.1 μg of GLP-1/ 1, 2, 3 μg of GLP-1  Intra-NAc shell 0.025 or 0.1 μg of GLP-1 | Exp1: Lateral ventricle (LV) injections of 1. 0, 0.033, 0.15, 0.3, 1, or 3g of GLP-1 2. 0, 2.5, 5, 10, or 20 g of Ex9 Exp2: NAc core injections of 1. 0.025 μg of GLP-1 2. 0.1 μg of GLP-1  3. 1, 2, 3 μg of GLP-1  4. 0.5 μl saline  Exp3: 1. Intra-NAc core saline 2. 0.1 μg of GLP-1 3. 1 ml of 0.6 M LiCl  4. 0.02% saccharin | Body weight Food intake measured 1, 2, and 24 h after injection Conditioned taste aversion (CTA)/Retrograde tracing into the NAc core IHC for GLP-1 levels c-Fos | Retrograde injections into the NAc core labeled caudal level NTS neurons and these NTS neurons were all GLP-1 positive; therefore, these results identified a strong GLP-1 projection to this forebrain region 1 and 3 μg intra-LV GLP-1 injections reduced food intake at 1 and 2 h (but not 24 h); lower doses had no effect 20 μg Ex-9 increased food intake at 1, 2, and 24 h; lower doses did not affect chow intake 0.025 and 0.1 μg intra-NAc core GLP-1 injections reduced food intake at 1, 2, and 24 h, so even subthreshold intra-NAc core infusions resulted in decreased food intake The core of NAc was more relevant to the GLP-1 action then the shell because of the intra-NAc shell injection of the same doses of GLP-1 had no effect  Intra-NAc core 3 μg Ex-9 increased food intake at 2 h and there was a trend of increased food intake at 24 h, meaning GLP-1 release from neurons at this site played a limiting role in the consumption of food There was no effect on body weight in both GLP-1 and Ex-9 injections Intra-NAc core GLP-1 (0.1 μg) injections increased the number of c-Fos-positive nuclei in the NAc core region NAc core was a second site for GLP-1’s food consumption effects without induction of CTA, so anorexia observed after NAc GLP-1 injections was not due to viscerosensory stress, but due to increased satiety |
| (Dailey et al., 2012) | Sprague Dawley rats (287.5 ± 12.5 g) | High fat (6 h) and chocolate (2 h) entrainment on separate groups | 1. Standard chow meal entrained 2. Standard chow ad libidum 3. High-fat diet (HFD) meal entrained 4. High-fat diet (HFD) ad libidum 5. Chocolate entrained 6. No chocolate meal given | Food anticipatory activity (FAA) 4 h before mealtime/Blood glucose levels Plasma ghrelin and insulin levels Plasma GLP-1 levels  Plasma collected from rats killed at 90, 60, 30 min prior to mealtime | The chocolate-entrained group did not show the same anticipatory increases in ghrelin and GLP-1 seen in the chow- or high-fat-entrained animals In all entrainment groups, an increase in glucose and a decrease in insulin occurred before mealtime Elevations in ghrelin and GLP-1 plasma levels were not necessary for the FAA to occur; thus, no correlation between these systems could be deduced Anticipatory changes in peptides might be due to food restriction or decreased body weight compared with the ad libitum control animals and not by different meal entrainments GLP-1 increased before meal feeding in rats The amount of food eaten or the energy status of the animal might be more important than the feeding pattern for the preprandial rhythm in ghrelin and GLP-1 Earlier increase in glucose in the groups that were expecting a greater caloric load might be correlated with the earlier onset and longer-lasting FAA response |
| (Mathes et al., 2012) | Male Sprague-Dawley rats | RYGB surgery All groups were tested in three conditions: water restricted, fasted, nondeprived Chow fed/Ex-9 (30 ug/kg) IP Ex-4 (1 ug/kg) IP | Exp12:  1. Sham + Vehicle 2. RYGB + Vehicle 3. Sham + Ex-9 4. RYGB + Ex-9 5. Sham + Ex-4 6. RYGB + Ex-4 Exp3: 1. Ex-9  2. Ex-4  3. Ex-4 + Ex-9 | Short-term brief-access licking test (while fasted pre-surgically and post-surgically and while nondeprived post-surgically and 5 h after injections/ another group while post-surgically or 15 min after injections both fasted and non-deprived) Body weight | Overall, sucrose lick scores of non-deprived rats were higher with Ex-9 than vehicle-injected rats/ however, this effect was small (15 min after injection) 0.3 M sucrose intake was decreased significantly with Ex-4 injection in fasted rats (30 min and 1 h after trial initiation) When Ex-9 and Ex-4 injected together, this effect was decreased with Ex-9 |
| (Alhadeff et al., 2012) | Male Sprague-Dawley rats | Ex-4 (0.025 μg and 0.05 μg, unilateral) into VTA, NAc core and shell 1 h after the onset of light cycle Ex-9 (10 μg) directed to either the VTA, NAc core or NAc shell immediately before the onset of the dark cycle | 1. aCSF 2. Ex-4 (0.025μg) 3. Ex-4 (0.05μg) | Cumulative sucrose intake (sucrose presented immediately after injections, assessed at 2 h, 5 h, and 24 h after injection) Modified chow or high-fat food intake (assessed at 1, 3, 6, and 24 h after injection) Pica response (14 h after injection) Body weight (24 h after injection)/Double immunohistochemistry for Fluorogold (monosynaptic retrograde tracer) and GLP-1  Immunoreactivity for PPG levels in VTA, NAc core, NAc shell | Neurons that express PPG in the NTS projected directly to the VTA, NAc core, and NAc shell Sucrose intake was reduced by intra-VTA and intra-NAc core (but not shell) Ex-4 (both subthreshold and above threshold doses) at 20, 30, 40, 50, and 60 min after injection  Ex-4 at both doses resulted in reduced body weight Both doses of Ex-4 into the VTA decreased HF diet intake at 6 and 24 h and increased chow intake at 3 h, along with 24 h reduced body weight gain, which indicated a shift in preference of highly-palatable foods 0.05 μg Ex-4 into NAc core decreased HF diet intake at 3, 6, and 24 h, while 0.025 μg only decreased HF diet intake at 24 h, along with 24 h body weight decrease 0.05 μg Ex-4 into NAc shell decreased HF diet intake at 6, and 24 h Ex-4 into NAc shell increased chow intake at 3 h 24 h of chow intake and body weight did not change by Ex-4 NAc (both core and shell) Intake of HF food was increased by intra-VTA (3, 6 h) and intra-NAc core (1, 3 h) Ex-9/ intra-NAc shell Ex-9 did not affect the intake of HF food Intra-VTA, intra-NAc core, and shell Ex-4 did not induce a pica response or decrease chow intake |
| (Dickson et al., 2012) | Male Sprague Dawley rats (250 g) | Partially food restricted/ lower levels of endogenous GLP-1/Ex-4 (0.3 μg/kg, 2.4 μg/kg) IP  Third ICV Ex-4 (0.03 μg, 0.1 μg, 0.3 μg, 1.5 g, 3.0 μg) Lateral ICV Ex-4 (0.2 μg) and Ex-3 (20 μg) Intra-VTA and -NAc Ex-4 (0.03 or 0.1 μg) Intra-VTA Ex-4 (0.01 μg) | 1. Vehicle 2. Ex-4 3. Third ICV vehicle 4. Third ICV Ex-4 5. Lateral ICV vehicle  6. Lateral ICV Ex-4 + vehicle  7. Lateral ICV Ex-4 + Ex-3 8. VTA Ex-4 9. NAc Ex-4 | PR operant conditioning at 10 min post-injection at 10, 30, 60, 120 min time points CPP at 10 min post-injection Motor activity (locomotor and rearing) for 1 h Pica response Body weight for 1 and 24 h Food intake for 1 and 24 h | Ex-4 IP. decreased lever pressing in the operant conditioning test and abolished preference in the CPP test ICV Ex-4 decreased the number of sugar rewards earned at most doses and at all time points Ex-3 reversed the Ex-4 induced decrease in food-motivated behavior At a dose subthreshold ICV, intra-VTA Ex-4 (0.03 and 0.1 μg) reduced food-motivated behavior, chow food intake, and body weight without affecting locomotor activity or rearing NAc Ex-4 (only at 0.1 μg) decreased operant responding and food intake less effectively than VTA and did not affect body weight NAc Ex-4 also reduced locomotor activity and rearing for a short amount of time VTA Ex-4 decreased operant responding for sucrose and food intake in satiated rats also Satiated rats that were more willing to work for sucrose at baseline (high responders) were more affected by VTA Ex-4 compared to less motivated rats (low responders) Neither VTA nor NAc Ex-4 treatments caused pica response |
| (Labouesse et al., 2012) | Male Sprague–Dawley rats (160-180 g) | SDA surgery: transection of the left dorsal vagal rootlets and dorsal esophageal vagal trunk resulting in complete vagal deafferentation below the diaphragm Food deprivation for 4 h before injections and 6 h before c-Fos/Ex-4 (1.0 and 0.1 μg/kg) IP | Exp1: 1. SDA surgery 2. Sham operation Exp2: 1. Ex-4 2. Bolus (1 ml/kg BW) | Cumulative food intake (0.5, 1, 2, 4 and 20 h) Meal patterns (first and second meal size and duration, inter-meal interval) Two-bottle choice paradigm (flavor avoidance learning)/IHC for c-Fos in NTS, DMX, AP, caudal VLM, PBN, DR, PVN, Arc, CeA, NAc | High dose (1.0 μg/kg) Ex-4 reduced 0.5 and 1 h food intake in both sham and SDA rats Low dose (0.1 μg/kg) Ex-4 decreased 0.5 and 1 h food intake in sham, but not SDA-operated rats Both Ex-4 doses decreased food intake in other time points regardless of surgery SDA rats tended to have decreased food intake regardless of Ex-4 Low dose Ex-4 decreased first meal size only in sham-operated rats First meal duration, second meal size or duration, inter-meal interval, and number of meals in the dark phase did not depend on the surgery Ex-4 induced a conditioned flavor avoidance in the SDA group but not in the sham-operated group/ indicating vagal afferents protected against Ex-4 mediated CFA Ex-4 caused activity in the AP, NTS, PBNle, CVLM, CeA, NAc and DMX regardless of surgery Ex-4 did not activate neurons in the PBN, DR, Arc Ex-4 caused activity in the PVN of sham-operated rats but not SDA rats |
| (Liang et al., 2013) | Adult, male Sprague Dawley rats (250-275 g at arrival and 638 g during the drug injection tests) | Ex-4 (1, 3.2, 10 µg/kg) IP | 1. Naltrexone (NTX) (0, 0.32, 1 and 3.2 µg/kg) 2. Ex-4 (0, 1, 3.2, 10 µg/kg) 3. NTX + Ex-4 | Short-term food intake Conditioned taste aversion (CTA) learning | NTX and Ex-4 decreased food intake dose-dependently NTX and Ex-4 combination decreased food intake and the amount of decrease was higher, which indicated an additive effect In the CTA test, NTX did not change support acquisition Both Ex-4 doses (1 or 3.2 µg/kg) and combinations of NTX and Ex-4 changed acquisition of CTA rapidly and robustly |
| (Dossat et al., 2013) | Naive male Wistar rats | Exp1: Sweetened condensed milk (SCM) Exp2: 0.25M sucrose Exp3: 0.10 sucrose Exp4: 0.1% saccharin Exp5: 3 ml IG 40% sucrose or saline/Intra-NAc core Ex-9 (3 μg on experiments 1-4 and 2 μg on experiment 5) 15 minutes prior to testing | Exp1-4: 1. Ex-9 2.Saline Exp5: 1. Vehicle + intragastric saline 2. Vehicle + intragastric sucrose 3. Ex-9 + intragastric saline 4. Ex-9 + intragastric sucrose | Body weight Food intake Licking microstructural variables | Ex-9 increased first meal size and decreased the number of meals of sweetened condensed milk, without affecting total licks Ex-9 increased 1st and 3rd meal size and total licks of 0.25M sucrose, without affecting the number/schedule of meals, satiety ratio, or within-burst interlick interval For 0.25M sucrose 1st meal, Ex-9 increased meal duration and initial lick rate, without affecting burst variables For 0.25M sucrose 2nd meal, Ex-9 increased burst size and duration and decreased the number of bursts For 0.25M sucrose 3rd meal, Ex-9 increased burst size and duration without affecting the number of bursts Ex-9 increased 2nd meal size and total licks of 0.10 sucrose, without affecting number/schedule of meals, satiety ratio, meal duration, number of bursts, or within-burst interlick interval For 0.25M sucrose 1st meal, Ex-9 increased the initial number of licks, burst size, and duration in the first segment of the meal For 0.25M sucrose 2nd meal, Ex-9 increased burst size and duration Ex-9 did not affect any variable for 0.1% nonnutritive saccharin, indicating the necessity of nutrients in the gut for the action of NAc GLP-1R Ex-9 reversed IG sucrose-induced decrease in size of first meal and burst size and duration in the first and third segment IG sucrose decreased the number of licking bursts and first meal duration regardless of Ex-9 IG sucrose or Ex-9 did not affect the number of licks in the first minute or within-burst interlick interval |
| (Mietlicki-Baase et al., 2013) | Adult male Sprague-Dawley non-obese (381.7 g, 11-14 weeks of age) rats | HFD (60% kcal from fat) during and for 2 weeks prior to testing/Intra-VTA Ex-9 (10 μg) unilateral Intra-VTA Ex-4 (0.05 μg) unilateral Ex-4 (3 g/kg) IP | Exp1: 1. VTA Ex-9 + Ex-4 or saline 2. VTA saline + Ex-4 or saline Exp2: 1. VTA CNQX (AMPA/kainate receptor antagonist) + VTA Ex-4 or saline 2. VTA MK-801 (NMDA receptor antagonist) + VTA Ex-4 or saline 3. VTA saline + VTA Ex-4 or saline | HFD food intake for 24 h Body weight at 24 h Meal patterns (meal size, number of meals) for 24 h/Immublotting in the VTA for TH at 15 min post-injection/Patch-clamp electrophysiological recordings in the VTA before and during Ex-4 application (1 μM) | Intra-VTA Ex-9 attenuated the IP Ex-4-induced decrease in HFD food intake and body weight Intra-VTA Ex-4 was found to reduce HFD food intake (from 3 to 24 h) by decreasing meal size and these reductions were suppressed by AMPA/kainate receptor antagonist CNQX NMDA-R’s were not involved in the food intake and meal size suppressive effects of intra-VTA Ex-4 Intra-VTA Ex-4 increased TH levels in the VTA compared to saline Ex-4 increased sEPSC frequency in VTA dopamine, without changing sEPSC decay, time, peak amplitude, and charge transfer |
| (Alhadeff and Grill, 2014) | Adult male Sprague-Dawley rats (250 –300 g) | HFD (45%, 60% kcal fat)/ 100-nl unilateral Ex-4 (0.025 or 0.05 μg) 100-nl unilateral mNTS injection of 0.025 μg of Ex-4 (0.025 or 0.05 μg) | 1. Vehicle 2. Ex-4 (0.025 μg) | HFD intake at 1, 3, 6, and 24 h post-injection Body weight at 24 h post-injection PR operant responding 3 h after injection CPP for palatable food 3 h after injection | mNTS-GLP-1R was found to decrease HFD intake and reduced PR responding for sucrose  mNTS-GLP-1R caused a reduction of CPP for palatable food mNTS-GLP-1R was observed to reduce chow intake and body weight, while not inducing pica response |
| (Alhadeff et al., 2014) | Adult male Sprague–Dawley rats (250–300 g) | HFD (45% kcal fat) for 5 days/Unilateral lateral parabrachial nucleus (lPBN) Ex-4 (0.025 or 0.05 μg depending on assessment) Ex-9 (10 or 20 μg) | Pica, chocolate pellets experiments:  1. Control  2. 0.05 μg Ex-4 HFD, activity: 1. Control  2. 0.025 μg Ex-4  3. 0.05 μg Ex-4 4. 20 μg Ex-9 | Body weight at 24 h HFD food (at 1, 3, 6, and 24 h), chow, and water intake (at 24 h) Pica response Operant lever responding Activity test/lPBN Fluorogold tracing  IHC for GLP-1 in NTS | 71.3% of GLP-1 producing neurons of the NTS monosynaptically projected to the IPBN ipsilaterally Cerebral aqueduct delivery of neither dose of Ex-4 nor Ex-9 affected HFD intake at all time points compared to vehicle Intra-lPBN Ex-4 decreased cumulative (6 and 24 h) and noncumulative (3-6 h) chow and water intake without causing pica, but it did not significantly reduce body weight Intra-lPBN Ex-4 decreased cumulative (6 and 24 h) and noncumulative (3-6 h) HFD and water intake, and body weight Intra-lPBN Ex-9 increased cumulative chow (6 and 24 h) and HFD (6 h) intake without affecting body weight Intra-lPBN Ex-4 decreased number of chocolate pellets earned and active lever presses Neither intra-lPBN Ex-4 nor Ex-9 significantly affected total distance traveled or total active time |
| (Yang et al., 2014) | Adult male Sprague Dawley rats (250 –275 g) | HFD or chow for 6 weeks Pair-fed groups were included (control for food intake and body weight)/Ex-4 (3.2 μg/kg) IP, twice daily for 9 consecutive days | 1. HFD +Ex-4 2. HFD +Vehicle  3. HFD + Pair-def 4. Standard chow (SC) + Ex-4 5. SC + Vehicle  6. SC + Pair-fed | Food intake daily (1, 2, 4, 8, and 24 h of intake) Body weight daily/Blood glucose, insulin, and leptin levels Agouti gene-related protein (Agrp), neuropeptide Y (NPY), proopiomelanocortin (POMC) expressions in the hypothalamic arcuate nucleus (ARC)  TH expression in the VTA Dopamine receptor (D1, D2) expressions in the NAc | Ex-4 decreased body weight and food intake in both SC and HFD/ this effect was seen earlier in the HF group Leptin and insulin levels were increased in HFD group/ long-term Ex-4 treatment successfully decreased these effects HFD decreased POMC but did not affect the expression of NPY or Agrp in the ARC Ex-4 increased POMC expression while decreasing NPY, and this effect was independent of the reduction of food intake and body weight  Mesolimbic TH and D1R gene expression were significantly decreased in chronic HFD rats/ Ex-4 and food restriction reduced these decreased expressions (only D1R changes reached significance) |
| (Mietlicki-Baase et al., 2014) | Adult male Sprague Dawley rats (375– 425 g) | HFD (60% kcal from fat) during and for 1 week prior to testing/Intra-NAc core injection of Ex-4 (0.05 µg) Bath application of 1µM Ex-4 for electrophysiological recordings | 1. NAc core AMPA/kainate receptor antagonist, CNQX (0.3 µg) or NAc core NMDA antagonist, AP-5 (1 µg) + vehicle 2. CNQX or AP-5 + Ex-4 3. Vehicle 4. Vehicle + Ex-4 | Body weight at 24 h Cumulative HFD food intake (1, 3, 6, and 24 h)/Fast-scan cyclic voltammetry for dopamine release measurements/Whole-cell voltage-clamp recordings Paired-pulse ratio (PPR) experiments | Ex-4 did not alter dopamine release in NAc core slices Ex-4 increased the frequency of NAc core MSN mEPSCs but did not affect kinetics or amplitude, indicating a presynaptic effect of GLP-1R activation Ex-4 bath application decreased the PPR of evoked EPSCs, further supporting a presynaptic effect by increasing the probability of glutamate release Ex-4 moderately suppressed the frequency of MSNs AP firing and slightly decreased (but significantly) resting membrane potential Intra-NAc Ex-4 decreased HFD intake and CNQX pretreatment reduced this effect Intra-NAc core Ex-4 reduced 24h body weight gain and CNQX reversed this effect AP-5 did not alter the anorexigenic effects of Ex-4 |
| (Anderberg et al., 2014) | Male Sprague−Dawley rats (250 g at the beginning) | Rats were food-restricted (10 g of chow) overnight/Ex-4 (0.2 μg/) i.c.v. | Exp1: 1. Food 2. No food Exp2: 1. Vehicle 2. SKF, D1 receptor agonist (2 μg) 3. SCH, D1 receptor antagonist (1 or 5 μg) 4. QNP, D2 receptor agonist (10 μg) 5. ETC, D2/D3 receptor antagonist (10 μg) 6. Ex-4 Exp3: 1. Vehicle + vehicle 2. Vehicle + Ex-4 (0.3 μg) 3. ETC (3.3 μg) + Ex-4 4. ETC + vehicle | Chow intake for 20 min at 1, 2, and 24 h Locomotor activity at 20 min after injection PR operant conditioning for sucrose at 20 min after injection/Amygdala dopamine turnover 30 min after injections or food intake DOPAC and HVA tissues concentrations 30 min after injections or food intake | Chow food intake and Ex-4 increased DOPAC and HVA levels along with amygdala dopamine turnover Operant responding for sucrose, chow intake, or locomotor activity was not changed by D1 receptor activation or D1 receptor blockade Food intake was decreased along with food-motivated behavior by D2 receptor activation and increased by D2 receptor blockade in the amygdala Food intake at all time points was decreased by central Ex-4 and D2/D3 receptor blockade reversed this effect  Ex-4 reduced operant responding for sucrose, but D2/D3 receptor blockade did not attenuate this effect |
| (Hansen et al., 2014) | Male Sprague-Dawley rats (8 weeks of age) for acute drug treatment Male Sprague-Dawley rats (6 weeks of age) for DIO study | Two-choice diet: rats fed standard rodent chow or HFHS diet for 17 weeks (diet-induced obese rats, DIO)/GLP-1 (0.4 mg/kg s.c.) Linagliptin (1.5 mg/kg) perorally or (0.5 mg/kg) s.c. Liraglutide (0.2 mg/kg) s.c. Acute or chronic treatment (bi-daily, 2 and 10 h into the light phase, for 28 days total) | Exp1: Acute treatment 1. Vehicle 2. Linagliptin 0.1 mg/kg 3. Linagliptin 0.5 mg/kg 4. GLP-1 0.2 mg/kg 5. GLP-1 0.4 mg/kg 6. Liraglutide  Exp2: Chronic treatment (DIO rats) 1. Vehicle 2. Linagliptin 0.5 mg/kg 3. Linagliptin 1.5mg/kg 4. GLP-1 0.4 mg/kg 5. Linagliptin 0.5 mg/kg + GLP-1 0.4 mg/kg 6. Linagliptin 1.5mg/kg + GLP-1 0.4 mg/kg 7. Liraglutide | Whole-body composition and abdominal fat analysis in DIO rats Cumulative food intake 6-20 h for acute treatment Chow, high-fat palatable diet (HPFC) and cumulative daily caloric intake for chronic treatment/Active and total plasma GLP-1 levels and DPP-IV enzyme activity of DIO rats ISH for CART, NPY, μ-opioid receptor, prepro-enkephalin, prepro-dynorphin, TH, dopamine transporter in the forebrain, midbrain, and hypothalamus of DIO rats | Linagliptin alone did not affect acute food intake at any time point and GLP-1 alone slightly and short-lastingly reduced food intake at 4 h after treatment A significant reduction in food intake was observed in combined linagliptin and GLP-1 in a dose-dependent manner until 6 h post-treatment Liraglutide alone significantly decreased food intake from 4 h post-treatment until the end of the 20th hour Both routes of linagliptin used in combined treatment affected the body weight loss equally The body weight was also reduced by liraglutide alone A marked increase in chow preference and decrease in HFHS diet intake was observed in both combined linagliptin and GLP-1, and liraglutide alone; however, this increase was more pronounced in the combined treatment Active and total plasma GLP-1 levels after 4 weeks of treatment were significantly higher compared to vehicle control and also GLP-1 alone in DIO rats Prepro-dynorphin mRNA levels in the caudate-putamen but not in the NAc were increased in linagliptin (s.c.) and GLP-1 combined in DIO rats, but this effect was not observed with liraglutide alone No significant differences were seen in other transcripts and regions between treatment groups |
| (Wright and Rodgers, 2014) | 10 adult male Lister hooded rats (beginning weights: Exp1 202.5 g, Exp2 215.4 g) | Exp1. Ex-4 (0.025, 0.25 and 2.5 μg/kg)  Exp2. Ex-4 (0.025 μg/kg and 0.25 μg/kg) | Exp1: 1. Vehicle 2. Ex-4  Exp2: 1. Vehicle 2. Ex-4 0.025 μg/kg + saline 3. Ex-4 0.25 μg/kg + 0.1 mg/kg NTX 15 min between injections | Latency for food location and eating for palatable mash 15 min after injections Frequency and duration of food intake, drinking, grooming, scratching, sniffing, and locomotion behaviors for palatable mash 15 min after injections Behavioral satiety sequence for 1 h of palatable mash 15 min after injections Weight gain post-treatment | Ex-4 significantly reduced food intake in a dose-dependent manner  The lowest dose of Ex-4 (0.025 μg/kg) failed to reduce food consumption and change eating behavior At 0.25 and 2.5 μg/kg doses, food intake, frequency, and consumption rate was decreased and these effects were more significant at 2.5 μg/kg Duration of eating bouts was increased, but there was no effect on the duration of eating at 0.025 μg/kg, but the total eating rate was observed to be declined dose-dependently Duration and frequency of sniffing, grooming and other locomotion behaviors and also resting periods were increased with Ex-4 dose-dependently Highest doses of both Ex-4 and NTX alone reduced eating behaviors considerably and produced an augmented dose-dependent anorectic effect NTX and Ex-4 combination failed to cause a more substantial decrease in food intake behaviors and degree of consumption relative to responses to Ex-4 and NTX alone |
| (Richard et al., 2015) | Adult, male Sprague-Dawley rats (180–250 g at arrival and 400 g during the drug injection tests) | Intra-NTS microinjection of Ex-4 (0.05 μg and 0.2 μg) | For CPP: 1. Ex-4 (0.05µg) injected rats 2. aCSF injected rats For RNA isolation and mRNA expression: 1. 0.2 µg Ex4 injected rats 2. aCSF injected rats | Palatable food-choice test (at 1,3, 6 h after injection) Pica test (at 1,3,6, and 24h after injection) Food induced operant conditioning  Conditioned place preference (CPP) (30 min after injection)/GLP-1 fiber detection TH, Drd1a, Drd2, Drd3, Drd5, Slc6a3, Gad1, Creb1, FosB mRNA expression | Intra NTS Ex-4 injection suppressed palatable food intake contrary to vehicle-treated rats Chow intake was notably decreased with the chow introduction to rats alone after Ex-4 injection in a dose-dependent manner Ex4 injection decreased body weight at 22h  No significant augmentation was seen in kaolin intake and there was no pica response Intra-NTS Ex-4 administration reduced the number of sucrose rewards gained, which implicated a decline in food-motivated behavior whereas there was no change in locomotor activity TH positive neurons and PPG positive neurons were colocalized at area postrema  TH encoding mRNA was fourfold, D2R in the VTA was twofold enhanced after intra-NTS injection; however, there was no alteration in FosB, Creb1, Gad1, D1aR, D3R, D5R expression in the VTA FosB, Creb1, Gad1, D1aR, D2R, D3R, D5R, and DAT did not significantly change in the NAc after Ex-4 injection |
| (Hsu et al., 2015) | Adult male Sprague-Dawley rats (320 - 450 g) | Exp1d. Western diet (41% kcal fat) Exp2a. 45 mg pellet (fat + sucrose)/Bilateral HPFv injections of Ex-4 (0.015, or 0.03 μg) Ex-9 (0, 1.25, 2.5, or 5, 10 μg) | Exp1a. HPFv or lateral ventricle (LV) Ex-4 (0.015, or 0.03 μg) or vehicle Exp1b. HPFv Ex-4 (0.03 μg) or vehicle Exp1c. HPFv Ex-(9-39) (1.25, 2.5, or 5 μg) or vehicle Exp1d. HPFv Ex-4 (0.03, or 0.06 μg) or vehicle Exp2a. HPFv Ex-4 (0.03 μg) or vehicle Exp2b. HPFv Ex-4 (0.03 μg) or vehicle Exp3. HPFv Ex-4 (0.03 μg) or vehicle, or 0.15 M LiCl or saline | Chow intake at 1, 3, 6, and 24 h after injections Body weight at 24 h post-injection Meal pattern immediately after injection Operant responding for food, PR reinforcement schedule 3 h post-injection CPP at 3 h post-injection Conditioned flavor avoidance (CFA) at 3 h post-injection/IHC for GLP-1 in the hindbrain GLP-1 levels in blood, CSF, and HPFv | Ex-4 delivered to HPFv, but not LV reduced cumulative food intake at 3, 6, and 24 h and body weight at 24 h HPFv Ex-4 decreased chow intake, meal size but did not affect meal frequency  10 μg HPFv Ex-9 increased food intake only at 6h HPFv Ex-4 increased preference for chow diet over the Western diet HPFv Ex-4 decreased the total number of active lever presses, the number of pellets earned, and operant response to food HPFv Ex-4 did not affect CPP or CFA GLP-1R axons were not detected in ventricular or dorsal HPF GLP-1R axons were found closely opposed to ventricular ependyma, including PVN, BNST, and subfornical organ FG injected into LV was found in some GLP-1R neurons in the NTS Active GLP-1 was found from highest to lowest in the serum, CSF and HPFv lysates |
| (Richard et al., 2016) | Female and male Sprague-Dawley rats (160–200 g) | Food-restricted (50% of normal intake) or non-deprived state/Ex-4 into left ventricle (LV) (0.1 or 0.3 μg/μL) ICV Ex-4 (0.3 μg/μL) | Exp1: Non-deprived or food-restricted 1. Vehicle 2. Ex-4 into LV Exp2: Food-restricted 1. Estrogen receptor antagonist, ICI (10 μg/μL) into LV + vehicle 2. Vehicle  3. ICI (10 μg/μL) into LV + Ex-4 4. Ex-4 Exp3: Food-restricted 1. Estrogen receptor-α (ERα) antagonist, MPP 2. Vehicle 3. MPP 4. Ex-4 | PR operant conditioning at 20 min after injection Chow (for 24 h) and palatable (peanut butter, for 1 h) food intake | Central GLP-1R activation in non-deprived animals was observed to decrease food-motivated behavior significantly more in females relative to males Not only desire for sucrose and peanut butter but also desire for chow intake decreased after 24 h in both sexes after Ex-4 In food-restricted animals, Ex-4 again decreased motivated eating behavior, but this time, sex was statistically insignificant ICI was observed to attenuate effects of Ex-4 on food behavior, in both sexes Ex-4 alone or in combination with ICI reduced peanut butter consumption in females, but not in males Ex-4 alone or in combination with ICI reduced chow consumption in males; this reduced consumption was only seen in Ex-4 in females MPP attenuated effects of Ex-4 on food behavior in both sexes |
| (Vogel et al., 2016) | Adult male Sprague-Dawley rats (200-250 g) | 2 μg GLP-1-estrogen (1.87 μg GLP-1, and 0.13 μg estrogen) s.c. 0.125 μg GLP-1-estrogen (0.117 μg GLP-1, and 0.008 μg estrogen) ICV 0.075 μg GLP-1-estrogen (0.07 μg GLP-1, and 0.005 μg estrogen into VTA, LH, NTS, and supramammillary nucleus (SUM) | 1. GLP-1  2. GLP-1-estrogen 3. Estrogen 4. Vehicle | Sucrose driven PR response in operant conditioning after 90 (s.c.) or 30 (central) min injections Chow intake for 1 and 24 h Body weight for 1 and 24 h Pica response Locomotor activity in the activity chamber at 30 min post-injection/SPECT-imaging of regional cerebral blood flow (rCBF) | Peripheral (s.c.) GLP-1-estrogen reduced food intake and body weight more than sole GLP-1 or estrogen (GLP-1, estrogen or vehicle alone did not reach significance) Peripheral conjugate injection reduced the number of sucrose pellets earned and active lever presses more efficiently than GLP-1 or estrogen alone After peripheral injections, pica responses were similar in all groups  ICV GLP-1-estrogen application had the same effect and did not cause malaise or locomotor impairment The SUM was found to be the target site of GLP-1-estrogen as increased blood flow was observed mostly in this area GLP-1 alone successfully reduced food reward in the VTA/ however, the conjugate was not effective  Intra-SUM GLP-1-estrogen reduced food reward and food intake more than GLP-1 or estrogen alone Intra-LH GLP-1-estrogen reduced food intake and body weight more than GLP-1 or estrogen-only Intra-NTS GLP-1-estrogen reduced food intake and caused a trend toward decreased body weight more than GLP-1 or estrogen |
| (Terrill et al., 2016) | Naive male Wistar rats | HFD (%60 fat)/Ex-4 (0.2 μg) i.c.v.  Unilateral, intra-LS Fluor 647-labeled Ex-4 (0.025 μg)  Intra-LV Ex-4 (0.025, 0.05, or 0.1 μg) 30 min before dark Intra-LS Ex-4 (0.025 μg) 30 min before dark Intra-LS Ex-9 (10 μg) 30 min before dark | Exp1-2: 1. Veh 2. Intra-LS Ex- 4 (0.01 or 0.025 μg) 3. Intra-LS Ex-9 (5 or 10 μg) Exp3: 1. Veh 2. Ex-4 Exp4:  1. Veh 2. Ex-9 Exp5-8: 1. Veh 2. Ex-9 3. Ex-4 | Chow intake at 1, 2, 4, and 20 h after injection Chow intake at 24 h after injection in other behavioral tests HFD (sucrose and corn oil) intake every 30 min for 2 h Pica response (kaolin intake) for 20 h Meal pattern analysis Elevated plus maze (EPM) PR operant responding for 2 h/Fluorescent immunostaining and fluorescently labeled Ex-4 in LS | Chow intake was reduced with doses of 0.05 and 0.1 μg at 20 h; however, 0.025 μg of Ex-4 did not differ from saline  Body weight was reduced by intra-LS Ex-4 as Ex-4 decreased overnight chow and HFD intake Chow intake was increased by intra-LS Ex-9 Average dark-phase meal size and average light-phase meal size were decreased after intra-LS Ex-4  Intra-LS Ex-4 did not induce pica response Sucrose intake and corn-oil emulsion intake were increased with intra-LS Ex-9 injection, but chow intake was not changed Breakpoint and total active lever presses and overnight chow intake were increased by intra-LS Ex-9 Total arm entries or entries into open arms were not affected by Ex-4, but Ex-4 significantly decreased the number of entries into closed arms in EPM Ensure preload significantly reduced PR responding In both the non-preload and Ensure conditions, intra-LS Ex-9 showed no effect on the number of sucrose reinforcers earned or total active lever presses  Chow intake was reduced by Ensure compared control no-preload condition Intra-LS Ex-9 increased 24-h total kilocalorie intake after Ensure load |
| (Alhadeff et al., 2017) | Adult male Sprague-Dawley rats (250-265 g) | Novel adeno-associated virus (AAV-GLP-1R) that has RNA sequences to knock down GLP-1R to NTS | 1. AAV-Control 2. AAV-GLP-1R | Food intake daily Body weight daily Meal patterns by feedometer 3 days for 2 weeks post-injection Operant responding for 45 mg sucrose pellets/Real-time PCR for GLP-1R mRNA levels | AAV transfected tissue showed 66.5% reduction in expression of GLP-1R compared to the control group in NTS tissues AAV-GLP-1R resulted in increased chow intake, dark cycle cumulative food intake, and meal size, but it did not increase the body weight and dark cycle meal number No difference in light cycle cumulative food intake or meal number was observed NTS AAV-GLP-1R increased the motivation to take the palatable food after training to receive 45 mg sucrose intake NTS GLP-1R mRNA expression negatively correlated with lever presses and reinforcers earned in the PR responding task |
| (Ong et al., 2017) | Adult male Sprague Dawley rats (250 - 265 g) | Chow or high-fat diet (HFD/ 45% kcal/fat)/Intra-PVT Ex-4 (12.5 ng, 25 ng, 50 ng)  Ex-9 (5 and 10 μg) | 1. HFD 2. Chow 3. HFD + Ex-4 4. Chow + Ex-4 | Chow and HFD intake at 1, 2, 4, 6, and 24 h post-injection Meal patterns CPP for HFD 4 h after injections Cue-induced reinstatement of sucrose-seeking behavior 4 h after injections PR responding for sucrose reward 4 h after injections Pica response/IHC for c-Fos, GLP-1, FLEx, and FG-labeled cells in the caudal NTS and PVT/Whole-cell patch clamp recordings for PVT-NAc projecting neurons | Intra-PVT Ex-4 decreased chow and HFD intake while intra-PVT Ex-9 increased it, independent of malaise Intra-PVT Ex-4 attenuated CPP for HFD and lever pressing for sucrose Food intake activated NTS PPG neurons, PVT projecting NTS neurons, and NTS PPG neurons that monosynaptically project to PVT PVT GLP-1 axons were found closely opposed to NaC projecting fluorescein Ex-4 labeled cells Ex-4 suppressed AP firing on PVT-to-NAc projecting neurons partly through synaptic mechanisms/ these inhibitory effects were blocked by Ex-9 (dose-dependently) Ex-4 induced suppression of AP firing was smaller and delayed when given with synaptic blockers (CNQX for glutamate receptors, PTX for GABA receptors) Ex-4 resulted in hyperpolarization of PVT-to-NAc neurons in the presence of synaptic blockers Ex-4 decreased frequency, but not amplitude, of spontaneous and miniature EPSCs in PVT-to-NAc (both core and shell) projecting cells |
| (Hsu et al., 2018) | Male Sprague–Dawley rats (320-450 g) | 1. Implanted bilaterally with vHP and mPFC-targeted cannulae, unilateral mPFC injection of AP-5 or vehicle, then vHP exendin-4 delivery  2. Short hairpin RNA targeting GLP-1R mRNA was cloned, packaged into an adeno-associated virus (AAV1), delivered bilaterally to the vHP (vHP GLP-1R KD group)/Intra-ventral hippocampus (vHP) injection of Ex-4 (0.03 μg) 200 nl AAV-GLP-1R shRNA (vHP GLP-1R KD group) | 1. mPFC AP-5 + vHP Ex-4 2. mPFC CNQX (0.3 μg) + vHP Ex-4 Bilateral vCA1 administration of:  1. Control (aCSF) 2. Control (AAV1-GFP) 3. AAV1-GLP-1R shRNA | Body weight Food intake Operant conditioning Differential reinforcement of low rates of responding (DRL)/FISH for GLP-1R in ventral hippocampal (vCA1) Immunohistochemistry (IHC) for PHAL, pNR2B,  Anterograde neural pathway tracing from the vHP field CA1 and AAV1-GFP | All FG back-labeled vCA1 cell bodies co-expressed GLP-1R mRNA vHP GLP-1R to mPFC NMDA-R neural pathway regulated impulsive responding for palatable food, but not AMPA/kainate receptor signaling mPFC pretreatment with vehicle followed by vHP Ex-4 exhibited a significant decrease in food intake and body weight vHP GLP-1R KD rats tended to choose greater meal size with a compensatory decrease in meal frequency, as well as a significant increase in inter meal interval compared to controls vHP GLP-1R KD rats earned a significantly greater number of pellets across a 5-day training period in comparison to control AAV and aCSF control Ex-4 also significantly increased efficacy (pellets earned/ total number of levers pressed) in an inhibitory control task |
| (Maske et al., 2018) | Naive male (25-300 g at arrival) Female (175-200 g at arrival) Wistar rats | Intragastric (IG) nutrient infusion for sucrose (15 kcal) and Ensure (9.3 kcal)/Ex-9 (100 μg/kg IP) | Exp1&2: 1. Saline  2. IG infusion of 10 ml 40% sucrose solution  Exp 3: 1. Saline  2. IG infusion of 10 ml Original Chocolate Ensure  Exp4:  1. 0.9 % saline 2. 1.46 % saline 3. 3.415% saline Exp5:  1. Vehicle  2. Ex-9 (100 μg/kg)  3. DVZ (0.5 mg/kg)  4. 10 ml of 0.9% saline  5. IG infusion of 10 ml Original Chocolate Ensure | Progressive ratio (PR) schedule of reinforcement Body weight  Overnight chow intake Overnight water intake/Vaginal cytology to define the estrous cycle in female rats | IG infusion reduced active lever presses, breakpoint, and number of reinforcers earned, which can partly be mediated by a decrease in motivation to get food in both male and female rats When released in response to IG infusion, GLP-1 might play a role in this effect in males IG infusion of saline was not sufficient to reduce motivation for food, so it is the caloric content of the infusion that decreased the motivation for food Food deprivation did not alter the effect of IG nutrients to reduce motivation for food  The gastrointestinal capacity did not play a role in the suppression of PR responding or breakpoint  IG infusion of Ensure decreased active lever presses, breakpoint, and number of reinforcers, meaning reinforcer, and the IG nutrient didn't have to be the same The suppressive effect of Ensure was blocked by the Ex-9 treatment in males but not females, measured by active lever presses and reinforces earned, without affecting the chow intake Devazepide, a CCKa-R antagonist, treatment failed to influence the reducing effect of IG infusion on food motivation in both males and females |
| (López-Ferreras et al., 2018) | Male and female Sprague-Dawley rats (5 weeks old) | GLP-1R KO in LH Ex-4 (0.05 μg and 0.15 μg) Ex-9 (10 μg) | Exp1: 1. Ex- 4 (0.05 μg) 2. Ex-4 (0.15 μg) 3. aCSF Exp2: 1. Ex-9 (10 μg) 2. aCSF Exp3: 1 h per day for one week, every other day 1. Fat diet  2. Chow diet  3. Sucrose diet | Progressive ratio operant conditioning for sucrose at 20 min after injection Food seeking with head-pokes into the chamber Food intake at 1 and 24 h after operant conditioning test Food intake and body weight daily (GLP-1 KD mice) Intraperitoneal glucose tolerance test/Fasting blood glucose analysis  qPCR for IL6, IL1, melanin-concentrating hormone (MCH), neurotensin, orexin expressions Viral tract tracing In situ hybridization (ISH) | Intra-LH Ex-4 (0.05 and 0.15 μg) significantly decreased food-motivated behavior (both reduced number of sucrose rewards earned and number of active lever presses) in male rats Intra-LH Ex-4 (0.05 and 0.15 μg) decreased food intake at both 1 and 24 h in males Only 0.15 μg intra-LH Ex-4 injection resulted in decreased sucrose rewards earned and food intake (at 24 h) in females Intra-LH Ex-4 decreased food reinforcement only in the estrus phase and not in metestrus/diestrus and reduced food intake at 1 and 24 h was more robust in the estrus phase as well Bilateral LH Ex-9 injections increased food reward earned, active lever presses, and food-seeking in males and not in females Bilateral LH Ex-9 did not affect food intake at 1 and 24 h GLP-1R KO increased operant responding for food reward and food intake in non-restricted male rats but not in females LH-GLP-1R activation reduced orexin expression but only in female rats during the estrus phase IL1, IL6 expressions significantly increased in male rats after LH-GLP-1 Ex-4 injections, and this increase in IL6 was only seen in consumption of sucrose but not chow or lard Expression of MCH (an orexigenic LH neuropeptide) was reduced by LH-GLP-1R activation in both males and females (during all female cycles), which could be associated with both the reinforcement and adiposity effects of LH-GLP-1R activation Female brains more readily compensated for the decreased LH-GLP-1R signal ILs and MCH were affected in both sexes, where reduction in orexin and neurotensin detectable only in females, specifically in the estrus phase |
| (Howell et al., 2019) | Male Sprague-Dawley rats | Ad libitum access to standard rodent chow and water were given to mice/Ex-4 (IP, 0.1–1.0 μg/kg/ VTA, 0.01–0.1 μg) | 1. VTA Ex-4 + 300 pmol ghrelin 2. VTA Ex-4 (0.01–0.1 μg) + saline vehicle | Operant responding for food reward with banana-flavored sucrose pellet reinforcers | The operant responding for food reward was increased by ghrelin administration into the VTA, and the peripheral injection of Ex-4 decreased this effect Both IP and VTA Ex-4 injection caused a significant decrease in operant responding for food reward that was constructed by ghrelin administration |
| (Jones et al., 2019) | Male and female Sprague-Dawley rats | Standard low-fat chow and water ad libitum for 2 weeks Some rats received a Western diet (high-fat (42%) and high-carbohydrate (39%) WD)/Liraglutide (10 μg/kg) IP | 1. Chow + saline 2. Chow + liraglutide 3. Western diet (WD) + saline 4. Western diet (WD) + liraglutide | Body composition and body weight at terminal training (before diet and drug) Transfer cue training: 12 daily, 4 min trials with a 10 s clicker that ended with the delivery of sucrose pellets (C+), except on trials where the clicker was presented without the sucrose reward (C-) sFN training: 10 second tone (3000 Hz) which ended with sucrose reward at the end of each 4 min trial (T+), except on trials where the tone was preceded by the presentation of a 4 min light (T-) and no sucrose reward followed Probe test began 4 d and 12 d after diet and drug initiation Mean nose pokes | Liraglutide did not show any significant effect on body weight; however, the body fat gain of liraglutide injected rats were significantly lower compared to saline-injected rats After the transfer cue training, the rats responded the Target+ (T+) trials more in comparison to non-target trials Mean nose pokes of rats treated with liraglutide were lower than rats treated with saline only on C- trials where there were not different in C+ trials Mean nose pokes of rats treated with chow liraglutide were lower than rats treated with chow saline in both T+ and T- trials Mean nose pokes of rats treated with WD + liraglutide were lower than rats treated with WD + saline in both T+ and T- trials Both sex and diet type were independent of the effects of liraglutide These results showed that liraglutide successfully enhanced the effect of inhibition to suppress responding to a reward cue/ However, it did not have a significant impact when the inhibitory cue was absent |
| (López-Ferreras et al., 2019) | Male and female Sprague-Dawley rats (3 weeks old) | High fat and sugar choice diet (HFHS) (a choice of lard, 30% sucrose solution, and chow)/Ex-4 into the supramammillary nucleus (SuM) (0.01 and 0.03 μg) Ex-9 (10ug) GLP-1R knockdown in the SuM, AAV-GLP-1R-shRNA | Exp1: 1. Male 2. Female Exp2: 1. Vehicle 2. 0.01 μg Ex-4 3. 0.03 μg Ex-4 Exp3: 1. Vehicle 2. Ex-9 Exp4: 1. Vehicle 2. Ex-4 Exp5: 1. Control 2. GLP1rKD | Operant response testing  Locomotor activity Chow, high caloric food intake (1 and 24 h) Body weight (24 h)/IHC for mCherry Neural tract tracing Blue-fluorescent DNA stain for cell count RNAscope Multiplex Fluorescent kit In situ hybridization for GLP-1R qPCR for GLP-1 mRNA expression Hematoxylin and eosin staining Adipose tissue collection | SuM bundle of fibers innervated the LH and dense GLP-1R mRNA expression was seen throughout the SuM fibers that projected to LH in both males and females SuM Ex-4 reduced ad libitum intake of chow in both male (both doses) and female rats (only the higher dose)/ along with reduced body weight at 24 h SuM Ex-4 reduced high-calorie food intake in both males and females SuM Ex-4 reduced the number of sucrose rewards and food-seeking behavior only in male rats SuM-Ex-4 did not change food-motivated behavior in females at all cycles Intra-VTA Ex-4 reduced motivation and food-seeking in females Food-seeking and adiposity in obese male rats was increased by LH, VTA, SuM GLP-1R KD without altering food intake, body weight, or food motivation in lean or obese, females were not affected as much as males in SUM, but they were similar in VTA injections |
| (Gabery et al., 2020) | Diet-induced obese (DIO) mice and rats | High-fat diet/Semaglutide (1, 3, 10, 30,100 nmol/kg/day) s.c. VitoTag750-S-labeled semaglutide (acute: 6 hours i.v., steady-state: 5 days, once daily s.c.) | Exp1: 1. Vehicle 2. Semaglutide Exp2: 1. Vt750-semaglutide 2. Vt750-liraglutide | Body weight, fat mass Food intake Palatable food preference Calorimetry/Fluorescent immunostaining and fluorescently labeled semaglutide and liraglutide Whole-brain c-Fos, GLP-1R immunoreactivity IHC for GLP-1R, SST, TH, CART, MCH, orexin, prolactin-releasing hormone (PrLH) , p-CREB qPCR Connectivity analysis/Light sheet fluorescence microscopy Electron microscopy | In DOI mice, semaglutide reduced food intake, body weight and fat mass dose-dependently In DOI rats, semaglutide transiently reduced food intake and decreased body weight dose-dependently In DOI mice, semaglutide significantly decreased chocolate intake compared to vehicle, but total chow intake was similar between groups Semaglutide targeted GLP-1R positive neurons in hypothalamus and hindbrain which were colocalized with other genes involved in control of appetite (CART, SST, TH) Semaglutide induced neuronal activation overlapped with neuronal pathways involved in meal termination controlled via lateral parabrachial nucleus In DOI rats, semaglutide increased PrLH and TH levels in area postrema |
| (Pierce-Messick and Pratt, 2020) | 16 male Sprague-Dawley rats | Ex-4 (0.05, 0.10 μg per side) intra-NAc core Ex-9 (2.5, 5.0 μg per side) intra-NAc core | Exp1: 1. Ex-4 2. Ex-4 + DAMGO (μ-opioid receptor agonist) Exp2: 1. Ex-9 2. Ex-9 + DAMGO | Food and water intake Locomotor activity | DAMGO significantly increased food intake and this effect was reduced by both doses of Ex-4 DAMGO enhanced ambulation, rearing, and locomotion and were blocked by Ex-4 DAMGO and Ex-9 combination significantly increased the length of food intake Ex-9 did not change the DAMGO induced ambulation and locomotor measurements |
| (Konanur et al., 2020) | 22 Long Evans rats | Fiber photometry surgery/Ex4 (0.05, 0.1 μg) into lateral ventricle | 1. Vehicle  2. Ex-4 | Licking behavior/IHC for GFP and TH Fiber photometry for measuring the activity of dopamine neurons | Ex-4 decreased the sucrose directed licking behavior and cue evoked dopamine activity in the VTA Sucrose directed behaviors was found to be associated with magnitude of cue evoked dopamine activity |
| (Vestlund et al., 2020) | Male Wistar Rcc Han rats (160-190 g) | Ex-4 (1.2 μg/kg) i.p. Ex-4 (0.05 μg per side) NAc shell Liraglutide (0.1 mg/kg) s.c. Dulaglutide (0.1 mg/kg) s.c. | Exp1: 1. Ex-4 2. Liraglutide 3. Dulaglutide Exp2: 1. Intra-NAc shell Ex-4 | Montoya staircase test (measures skilled reach foraging for sucrose pellets) Skilled reach performance test Body weight Rotarod test/Ex vivo field potential recordings Whole cell recordings | Ex-4 and liraglutide significantly decreased motivation for consumption of sucrose pellets in rats with acquired skilled reach performance (trained) but this was not seen with dulaglutide Intra-NAc shell Ex-4 also significantly reduced motivation in trained rats Ex-4 and liraglutide suppressed evoked field potentials in NAc shell Dulaglutide increased learning of skilled reach foraging (increased sucrose pellet consumption and better success rate) in untrained rats but this was not seen with Ex-4 or liraglutide |
| **1.b. Mice** | | | | | |
| (Wang et al., 2015) | Phox2b-Cre BAC transgenic mice | HFD (45 kcal%)/Chemogenic activation of GLP-1 expressing neurons in NTS by clozapine-N-oxide (CNO) injection Ex-4 (2.4 μg/kg, IP. and 0.24 μg/kg for intracranial) Exn-9 (8.4 μg/kg) IP | Exp1: 1. Control  2. CNO Exp2: 1. Control 2. hM3Dq (activates neuronal burst firing) 3. hM4Di (inhibits neuronal firing) Exp3:  1. Control  2. Ex-4 | Chow and HFD intake at 2, 5, and 24 h post-injection Body weight/IHC for TH, vGlT1, vGLuT2, and GLP-1 levels Retrograde labeling of VTA-to-NAc medial-shell-projecting neurons/Electrophysiological recordings from adult mouse midbrain | Highly palatable HF food intake was decreased by activation of NTS GLP-1 neurons by releasing GLP-1 GLP-1 release into the VTA caused decreased HF food intake Ex-4 application TH+ VTA-to-NAc projecting DA neurons suppressed AMPA-R-mediated EPSCs without changing NMDA EPSCs In the presence of TTX, Ex-4 significantly decreased the amplitude of DA neurons There were no changes recorded in mEPSCs of non-DA neurons |
| (Sirohi et al., 2016) | GLP-1R KDNestin mice  FLOX mice | Ad libidum chow or HFD/GLP-1R KD Nestin (GLP-1R selectively abolished from CNS) Ex-4 (30 μg/kg) IP | 1. Saline 2. Ex-4 | Locomotor activity Food intake (HF or chow) after 21 h of deprivation Body weight each hour for 2 h | Ex-4 completely blocked chow intake in both groups at 2nd hour and HFD intake in FLOX mice entirely and only to a certain extent in GLP-1R KD Nestin mice Locomotor activity did not change in both groups after Ex-4 No weight change was detected in both groups |
| (Yamaguchi et al., 2017) | C57/BL6J male mice (9 weeks old) | Binge-like sucrose overconsumption model: 7–8 day acclimation period Pre-training, ‘limited access’ training, and post-training phases In all three phases, 0.5 M sucrose, water and chow were provided daily In 'limited access' training (10 days), only water was given at night without chow In the pre and post-training, chow and water was given between 17:00-9:00, but sucrose was not/Lower GLP-1 group (500 nmol/kg IP BW) and higher GLP-1 group (1000 nmol/kg IP BW) | 1. Saline 2. Lower PYY (12.5 nmol/kg BW IP) 3. Middle PYY (25 nmol/kg BW IP) 4. Lower CCK-8 (2 μg/kg BW IP)  5. Higher CCK-8 (4 μg/kg BW IP) 6. Lower GLP-1 (500 nmol/kg BW IP) 7. Higher GLP-1 (1000 nmol/kg BW. IP) | Taste aversion conditioning performed at day 11 Sucrose and saccharin intake (pre-training, day 1, 10, 11)/PYY, GLP-1, CCK-8 levels | Exogenous PYY, CCK-8, and GLP-1 (both high and low doses) suppressed the consumption of sucrose compared to saline injections Higher doses of PYY, CCK-8 and GLP-1 did not result in conditioned taste aversion for saccharin, indicating these molecules did not cause visceral distress |
| (Mella et al., 2017) | 8-12 weeks old BALB/C male mice (20-25 g at arrival) | Cafeteria (CAF) diet (chow ad libidum, rotation of 10 sweet and 10 savory foods) or chow diet (ad libitum chow) for 12 weeks/Ex-4 injection of (0.1, 1, 5, and 25 μg/kg) IP | 1. CAF diet  2. Control diet | Preferred food intake during weeks 1 to 8 of the CAF diet Chow intake at 1, 3, 6, and 24 hours post-injection Body composition analysis  Conditioned place preference (CPP) test for chocolate at 10 weeks Chow and kaolin intake 3 hours after injection/Plasma GLP-1 levels Hypothalamic GLP-1R mRNA expression | Ex-4 non-significantly decreased chow intake at 1, 3, and 6 hours but not at 24 hours post-injection Mice fed the CAF diet showed higher weight gain, fat mass and developed preference patterns of palatable food over time CAF diet mice had decreased anorectic effect of Ex-4 on blocking of novel palatable food intake While there was palatable food present, the effect of Ex-4 decreased in terms of food inhibition Both CAF and chow diet mice showed a CPP for chocolate and Ex-4 only successfully blocked this effect on chow diet mice Mice fed the CAF diet showed a decrease in GLP1R mRNA expression in the hypothalamus compared to chow mice, but did not change active plasma GLP-1 levels Ex-4 decreased chow intake, but it did not increase kaolin intake Ex-4 doses used did not produce malaise |
| (Williams et al., 2018) | Naïve male C57Bl6J mice (22 g) | Cre-expressing mice HFD (60% fat) for 2 weeks prior to injections/Intra-BNST GLP-1 (0.1, 0.3, or 1.0 μg) Intra-BNST and intra-NAc Ex-9 (3 or 10 μg) | 1. Chow diet, intra-BNST GLP-1 (0.1, 0.3, or 1.0 mg) or saline 2. Chow diet, intra-BNST Ex-9 (3 or 10 μg) or saline 3. HFD, GLP-1 + saline or Ex-9 + saline | Food intake at 1, 2, 4 and 22 h Body weight daily Restraint stress (30 min)/Fourth generation neuroanatomical tracing: Distribution of PPG axons, NTS-PPG neuron projections to BNST/BNST electrophysiology Resting fire frequency recordings | NTS-PPG neuronal projections to BNST and other areas were similar with the YFP-PPG projections/ which showed strong projections between these regions Intra-BNST GLP-1 decreased chow intake at 1,2 and 4 h at all doses dose-dependently while Ex-9 increased it Intra-BNST GLP-1 less effectively suppressed feeding in HFD at 1 and 2 h (more significant at higher doses) and Ex-9 did not change intake in HFD mice Intra-NAc Ex-9 did not affect chow intake Intra-BNST Ex-9 attenuated restraint stress-induced hypophagia as food intake was higher at all time points compared to vehicle 100 nM GLP-1 reversibly depolarized or hyperpolarized BNST neurons, which was opposite to the response when dopamine was applied |
| (Terrill et al., 2019) | Naive male and female C57Bl6J mice or transgenic mice | Transgenic mice that express the YFP variant under the control of mGLU on a C57Bl/6 background Exp2: Large meal of chocolate Ensure 15 min prior to dark onset for 20 days (training period), access to Ensure for the 15 min just before lights out (experiment days)/Intra-LS GLP-1 (0.3 and 1.0 μg) i Bilateral intra-dorsal subregion of LS (dLS) GLP-1 (1.0 μg) Intra-LS Ex-9 (3 and 10 μg)  Bilateral intra-dLS Ex-9 (10 μg) | Exp1: 1. Intra-LS saline vehicle  2. Intra-LS GLP-1 (0.3 and 1.0 μg) 3. Intra-LS Ex-9 (3, 10 μg) Exp2-4 1. Intra-LS saline vehicle  2. Intra-LS Ex-9 (10 μg) Exp5: 1. Bilateral intra-dLS saline vehicle 2. Bilateral intra-dLS GLP-1 (1.0 μg) 3. Bilateral intra-dLS Ex-9 (10 μg) Exp6: 1. Saline vehicle 2. GLP-1 (1.0 μg) 3. Ex-9 (10 μg) | Body weight  Cumulative chow intake for 1, 2 and 4 h after dark onset Total session lick counts for sucrose daily (120 min) FR on operant responding 45 min after injections Meal patterns (meal duration, ingestion rate)/GFP immunoreactivity in hindbrain PPG neurons | Chow intake of mice was reduced (both in the dark phase and overnight intake) with intra-LS GLP-1 at subthreshold doses Intra-LS GLP-1 with Ex-9 (at subthreshold doses) did not change chow intake Ensure intake (large meal) before test sessions decreased subsequent chow intake and this effect was reserved with intra-LS GLP-1R Ex-9, but it did not affect body weight In the saline group, the acute stress significantly decreased food intake, but there was no effect of Ex-9 to the food intake during acute stress Whole intra-dLS GLP-1 decreased active lever presses; they were increased with bilateral dLS Ex-9 in operant responding (this effect remained even after mice were returned to ad libidum food intake) Lick rate during the first minute of the meal was suppressed by intra-dLS GLP-1 in ad libidum fed mice Intra-dLS GLP-1 suppressed burst number, whereas Ex-9 increased it, but there was no effect on burst size and duration After dLS GLP-1, the ingestion rate was decreased significantly |
| (Decarie-Spain et al., 2019) | 8-week-old C57BL/6J male mice | HF-HS for 12 weeks Withdrawal: HF-HS for 6 weeks, washout for 2 weeks (treatments)/GLP-1/Dexa (100 nmol/kg) s.c. GLP-1 analogue (100 nmol/kg) s.c. Daily for 11 days (last 2 weeks of HF-HS diet) For 3 weeks on operant conditioning tests | 1. Saline  2. GLP-1/Dexa 3. GLP-1 analogue alone 4. Pair-fed | PR operant responding Forced swim test (FST) EPM Open field test (OFT) Novel object recognition (NOR)/Quantitative PCR for D1r, D2rsh, d2rlg, Th, Dat, Dor, Kor, Mor, Gr, Mr in NAc core and shell | GLP-1/dexa significantly decreased food-motivated behavior for 20 hours, whereas GLP-1 analogue only showed a transient reduction (4 hours) in motivation, but the vehicle did not have a significant change in food motivation After withdrawal from the HF-HS diet, the mice which were treated with GLP-1/dexa showed less motivation for food reward comparison to mice treated with vehicle GLP-1/dexa decreased the expression of reward-related genes in the NAc such as D1r, D2rlg, Kor, Gr, whereas others were also reduced but non-significantly  GLP-1/dexa induced weight loss without changing anxiety and depressive-like behaviors in HF-HS diet mice |
