# Supplementary material for: Can GLP-1 Be a Target for Reward System Related Disorders? A Qualitative Synthesis and Systematic Review Analysis of Studies on Palatable Food, Drugs of Abuse, and Alcohol
Source: Front Behav Neurosci. 2021 Jan 18;14:614884. doi: 10.3389/fnbeh.2020.614884 (PMC7848227; doi:10.3389/fnbeh.2020.614884)
Supplement: Supplementary file 2 [file Table_2.DOCX]

| Supplementary Table 2. Preclinical studies on GLP-1 and drugs of abuse | | | | | |
| --- | --- | --- | --- | --- | --- |
| ***Name of First Author/ Publication Year*** | ***Mouse/ Rat Line & Age or weight*** | ***Definition of the Model/Application of GLP-1 agonist (dose and method)*** | ***Experimental Groups*** | ***Assessments*** | ***Main Findings*** |
| **1. Cocaine & amphetamine & opioids** | | | | | |
| **1.a Rats** | | | | | |
| (Lautar et al., 2005) | Exp1: 7 male Sprague-Dawley rats (225-250 g) Exp2: Diabetic homozygous db/db mice (20-25 g) 5-week-old Exp3: CDI male mice (20-30 g) Exp4-5: 10 mice male Rj NMRI (23–27 g) | Genetically diabetic db/db mice/A potent DPP IV inhibitor, 1-(2-Amino-3-methyl- butyryl)-azetidine-2-carbonitrile (AMAC) | Exp1:  1. AMAC 2. DAMAC (orally at 50 mg/kg) Exp2: After 45 min, oral glucose administration (1.5 g/kg) 1. AMAC (100 mg/kg)  2. Vehicle  Exp3: 1. AMAC/DAMAC (50 mg/kg, 1 mL/kg) 2. Vehicle Exp4: After 30 min, oral mescaline (50 mg/kg) 1. AMAC (10 mg/kg) 2. DAMAC (10 mg/kg) 3. Saline 4. Clozapine (2 mg/kg) Exp5: After 30 min, d-amphetamine (3 mg/kg) 1. AMAC (10 mg/kg) 2. DAMAC (10 mg/kg) 3. Saline 4. Haloperidol (0.25 mg/kg) | Locomotor activity every 5 min for 2 h Mescaline-induced scratching behavior Amphetamine-induced hyperactivity/Plasma and brain levels of AMAC and DAMAC  HPLC/MS-liquid chromatography Accu-Check Advantage strips for whole blood analysis Radioligand-binding and transport assays for 20 receptors involved in schizophrenia pathology Glucose tolerance test | Glucose tolerance in diabetic mice increased after acute and chronic AMAC suggesting that it behaved as a DPP IV inhibitor in vivo Both AMAC and DAMAC did not change locomotor activity After oral administration of mescaline, scratching behavior increased in mice; however, pretreatment with AMAC effectively decreased it in a dose-dependent manner AMAC was as effective as haloperidol in decreasing amphetamine-induced hyperactivity There was a trend towards dose-dependence with reductions in hyperactivity and only the highest dose had a significant effect DAMAC did not improve (decrease) mescaline-induced scratching and hyperactivity AMAC did not inhibit the binding of ligands in any of the receptors thought to be involved in schizophrenia |
| (Erreger et al., 2012) | Male Sprague–Dawley rats (275–300 g) | AMPH (2.78 mg/kg, IP) at time 120 min in the chamber/Ex-4 (30 μg/kg) IP at time 30 min in the chamber | 1. Saline + AMPH  2. Ex-4 + AMPH  3. Saline 4. Ex-4 + saline | OF Conditioned place aversion Locomotor activity | Ex-4 reduced basal and AMPH-induced locomotion Ex-4 did not result in anxiety-like behaviors and did not induce aversion |
| (Schmidt et al., 2016) | Male Sprague-Dawley rats (225–250 g) | Between-sessions, within-subjects design Intravenous cocaine (10 mg/kg) infusions on a FR5 schedule Once stable responding is achieved, switched to a PR schedule of reinforcement for 5 days/Ex-4 (0.005 and 0.05 μg) into the VTA immediately before the PR Viral-mediated GLP-1R KD | Exp1: 1. Vehicle 2. Ex-4 intra-VTA Exp2: 1. Vehicle 2. Corticosterone (0.05 and 0.5 μg) into the fourth ventricle 15 min before PR Exp3: 1. Vehicle + corticosterone 2. Ex-9 + corticosterone 3. Vehicle 4. Ex-9 + corticosterone Exp4: 1. Cocaine self-administration  2. Yoked saline controls Exp5: 1. AAV-GFP 2. AAV-GP-1R-shRNA | Total active lever responses and breakpoint Sucrose intake/GLP-1 mRNA expression levels  Plasma corticosterone IHC in the NTS for c-Fos and PPG 90 min after cocaine self-administration | Intra-VTA Ex-4 reduced cocaine-seeking at small doses (0.05 μg) that did not affect sucrose intake Cocaine-experienced rats showed greater plasma corticosterone levels compared to controls Corticosterone increased activity in PPG-positive cells in the NTS compared to the vehicle but did not affect total PPG-expressing cells Cocaine increased the activity of GLP-1 producing neurons in the NTS compared to controls while the total number of GLP-1-positive cells did not differ between treatments Fourth ICV corticosterone application reduced cocaine-seeking dose-dependently without affecting sucrose seeking, and intra-VTA Ex-9 blocked these effects  Knockdown of GLP-1 receptors significantly increased cocaine self-administration |
| (Reddy et al., 2016) | Male NMRI mice used for microdialysis and c-fos experiments Sprague Dawley rats (320–345 g) Adult male C57BL/6 mice | PC12 cells Local perfusion with cocaine (50 μM) for 30 min and five 30-min fractions were collected/Ex-4 (2.4 μg/kg) IP 30 min before killing 1and 10 nM GLP-1 for 20 min slice treatment 100 nM Ex-9 for 10 min slice treatment | 1. Vehicle 2. Arachidonic acid (AA) 3. Fe/Asc 4. Fe/Asc + AA | IHC for surface and total DAT and mApple High-speed chronoamperometry for DA release c-fos expression of GLP-1 30 min after treatment Surface and total DAT expression levels DA uptake | In the caudal and rostral lateral septum (LS), GLP-1R expressing cells were found opposed to DA terminals Ex-4 (at both doses) decreased LS activity and cocaine-induced DA release in the LS GLP-1R stimulation increased DAT surface expression and DA uptake in LS slices and GLP-1 antagonist blocks this effect on DA uptake In the LS, Ex-4 reduced retrograde messenger 2-AG and AA levels AA reduced DA uptake and DAT surface expression regardless of oxidative stress AA reduced DAT function more under oxidative conditions |
| (Fortin and Roitman, 2017) | Male Sprague-Dawley rats (350–425 g at the beginning) | 30 cocaine infusions (0.25 mg/0.1 mL, 3 seconds of infusion, 4 min of inter-infusion interval) paired with FSCV readings/LV Ex-4 (0.15 μg) immediately after 10th cocaine infusion | 1. LV Ex-4 (0.15 μg) in NAc core  2. LV Ex-4 (0.15 μg) in NAc shell 3. aCSF in NAc core 4. aCSF in NAc shell | Dopamine concentration changes Rate of dopamine reuptake /Fast-scan cyclic voltammetry (FSCV) recordings for phasic dopamine signaling in NAc shell and core | Cocaine increased phasic dopamine release in the NAc Cocaine infusion resulted in phasic dopamine changes in the NaC shell rather than the NAc core Suppression of cocaine-induced increase in dopamine concentration of NAc by Ex-4 was limited to the core The mechanisms controlling the extracellular concentration of dopamine remained stable and was not affected by the suppression of Ex-4 Both magnitudes of stimulated dopamine release and rate of dopamine uptake did not change by Ex-4 |
| (Hernandez et al., 2018) | Male Sprague-Dawley rats (225-250 g) | Acute priming injection of cocaine (10 mg/kg) Within-subjects design/Exp1. 3.0 µg/kg fluoro-Ex-4 IP 1 h before cocaine Exp2. 0.01, 0.1, and 0.2 µg/kg fluoro-Ex-4 IP 1 h before cocaine Exp3: Ex-9 (10 µg) 30 min before fluoro-Ex-4 IP Exp3. Ex-4 (0.005 and 0.05 µg) into VTA 10 min before cocaine | 1. Vehicle 2. Vehicle + Ex-4 systemic 3. Ex-9 to VTA + Vehicle 4. Ex-9 to VTA + Ex-4 systemic | Body weight change 24 h after each treatment Cumulative chow intake Cumulative number of meals Meal size Total active lever responses/IHC in VTA Quantitative RT-PCR for VTA GLP-1R and NTS PPG mRNA expressions | Ex-4 (3.0 µg/kg) reduced food intake and body weight  Low doses of Ex-4 (0.1, 0.2 µg/kg) attenuated cocaine-seeking in active lever presses but did not affect food intake, meal patterns, and body weight Intra-VTA Ex-9 blocked the attenuating cocaine-seeking effect of peripheral fluoro-Ex-4 GLP-1R activation directly in the VTA (0.05 µg/kg Ex-4) reduced cocaine-seeking dose-dependently but did not change sucrose seeking  Extinction (7 days) after cocaine self-administration resulted in diminished mRNA expression of PPG in the NTS |
| (Hernandez et al., 2019) | Male Sprague–Dawley rats (225-250 g) | Cocaine reinstatement: 10 mg/kg priming injection of cocaine after cocaine-taking behavior was extinguished and reinstatement test sessions Cocaine experimental rats: self-administer cocaine for 21 days (extinction phase: cocaine was replaced with saline for approximately 7 days)/Exp1: 0.1 and 0.2 μg/kg fluoro-Ex-4 (IP) 1 h before priming cocaine injection Exp2: Ex-4 (0.005 and 0.05 μg/500 nl) into NAc core and shell 10 min before priming cocaine injection | Exp1:  1. aCSF 2. Ex-4 systematic Exp2: 1. aCSF 2. Ex-4 to NAc core  3. Ex-4 to NAc shell  Exp3:  1. Yoked saline 2. Cocaine | Total active and inactive lever responses/IHC and PCR for GLP-1R expression in the NAc/Whole-cell current clamp | Cocaine seeking in rats was attenuated by peripheral fluoro-Ex-4 (at both doses), and this was localized in the NAc Cocaine seeking in rats was attenuated by direct Ex-4 into the NAc core (both doses) and shell (only the higher dose), and this administration did not alter the sucrose seeking in rats Medium spiny neurons in NAc core and shell of cocaine-experienced rats showed higher action potential frequency after Ex-4 Ex-4 on cocaine-experienced rats did not change sEPSC frequency or PPR of sEPSCs GLP-1R expression in the NAc showed no correlation with the extinction period (both at day 1 and 7) following cocaine self-administration |
| (You et al., 2019) | Long-Evans rats | Self-administer intravenous cocaine (1 mg/injection) in 4-hour sessions for 14 days on a FR schedule Extinction for 14 days of non-rewarding saline | 1. Saline 2. Cocaine | Growth hormone, AC-ghrelin, GLP-1, corticosterone, IGF-1, adiponectin, leptin, insulin, GIP, prolactin levels before and within the self-administration or extinction sessions | GLP-1 levels were doubled by cocaine, whereas corticosterone, IGF-1, and non-acetylated ghrelin levels were increased too (by 50%) Leptin, insulin, GIP, and prolactin levels were decreased by cocaine (by 40-70%) None of the parameters changed with saline  At the first day of extinction (day 15, saline), all of the markers mentioned above increased or decreased at the same extent as cocaine  At the 14th day of extinction (day 28, saline), the increases and decreases observed during cocaine were reversed |
| (Łupina et al., 2020) | Male Wistar rats (160-200 g) | Expression, acquisition, extinction, and reinstatement of morphine Morphine (5 mg/kg IP for place preference induction test, 1 mg/kg IP in the reinstatement procedure)/Linagliptin (10 and 20 mg/kg) IP during post-conditioning (60 min before testing), during conditioning (30 min before morphine injections) or extinction (60 min before testing, daily) | 1. Saline 2. Morphine 3. Morphine + linagliptin 10 4. Saline + linagliptin 10 | Morphine-induced CPP: pre-conditioning (day 1), conditioning (day 2-4), post-conditioning (day 5), extinction (day 6-10), reinstatement (day 11) Locomotor activity during expression and acquisition of CPP | Expression and morphine-induced CPP was inhibited by a single injection of linagliptin in saline rats, but no effect was observed in the time period, that was spent by animals at drug-associated compartment, during the post-conditioning phase and conditioning phase Morphine during the conditioning phase (acquisition) induced CPP in morphine-conditioned rats, as compared to saline rats and linagliptin (only in the lower dose, 10 mg/kg) reversed this effect The time that was spent by the rats at the morphine-paired compartment was increased by the challenge dose of morphine (1 mg/kg) A significant reinstatement of morphine-induced CPP was observed by the challenge dose of morphine in the morphine group as compared to the saline group  Linagliptin administration (only in the lower dose, 10 mg/kg) during the extinction phase inhibited reinstatement of rewarding effects of morphine by the challenge dose  Linagliptin (at both doses) administered during the extinction phase enhanced the extinction of morphine-induced CPP No changes in locomotor activity were observed in any period of the experiment |
| (Zhang et al., 2020) | Male Sprague-Dawley rats (250-300 g) | Systemic Ex-4 (0.3, 3.0 μg/kg)  Ex-4 (0.005, 0.05 μg/kg) intra-NAc shell | Exp1: 1. Vehicle 2. Ex-4 Exp2: 1. Vehicle 2. Intra-NAc shell Ex-4 | Body weight Food and water intake Oxycodone self-administration, reinstatement Tail-flick test | Both systematic and intra-NAc shell Ex-4 significantly decreased oxycodone self-administration, oxycodone and cue priming-induced reinstatement of oxycodone-seeking behavior dose-dependently without changing food intake Both systematic and intra-NAc shell Ex-4 did not change tail-flick latencies |
| **1.b Mice** | | | | | |
| (Graham et al., 2013) | Male wild-type C57Bl/6J mice (8 weeks) | Ex-4 (10, 30, and 100 ug/kg body weight) IP | 1.Ex-4 + Saline 2.Ex-4 + Cocaine HCl (20 mg/kg, IP) 3.Saline + Cocaine HCl  4.Saline + Saline | CPP at day 10 | Cocaine increased locomotor activity in CPP regardless of Ex-4 Only the Ex-4 alone pretreatment group showed reduced activity even at the lowest doses |
| (Egecioglu et al., 2013b) | Adult (8-12 weeks old) male NMRI mice (25 - 40 g) | Ex-4 (2.4 μg/kg) IP single injection | 1. Vehicle + vehicle 2. Ex-4 + vehicle  3. Amphetamine (2 mg/kg, IP) + vehicle 4. Ex-4 (at 10 min) + amphetamine 5. Cocaine (10 mg/kg, IP) + vehicle 6. Ex-4 (at 10 min) + cocaine | Locomotor activity 10 min after drug injection CPP/In vivo microdialysis for accumbal dopamine release collected every 20 min | Ex-4 attenuated the amphetamine- and cocaine-induced locomotor activity Ex-4 alone did not affect locomotor activity  Ex-4 attenuated amphetamine- and cocaine-induced accumbal dopamine release  Ex-4 alone did not affect accumbal dopamine release Ex-4 attenuated the amphetamine- and cocaine-induced CPP |
| (Harasta et al., 2015) | Glp-1r-/- and Glp-1r+/+ on C57BL/6J mice (2-4 months for behavioral testing) HEK293 cells | For behavioral tests: initial day with saline injections (baseline), cocaine (10 mg/kg IP) for days 1-5, 7 consecutive days drug and test free, priming injection of cocaine at day 12 for expression of sensitization/Glp-1r-/- and Glp-1r+/+ Viral vector-mediated Glp-1r gene delivery to the dLS of Glp-1r−/− mice | Exp1: 1. Glp-1r-/-  2. Glp-1r+/+ Exp2: 1. AAV-GFP for dLS + Glp-1r+/+ 2. AAV-GFP for dLS + Glp-1r-/-  3. AAV-GLP-1R-GFP into the dLS + Glp-1r-/-  Exp3: 1. Saline 2. Cocaine (10 mg/kg, IP) | EPM OF boxes for locomotor sensitization to cocaine (30 min sessions per test day) CPP for 30 min immediately after 14-days of last cocaine administration /Immunoblotting of GLP-1R mRNA expression RT-PCR IHC for GLP-1R ISH for GLP-1R expression in LS/Electrophysiological recordings of septal neurons | Almost all dLS cells expressed Glp-1r mRNA and most of those expressed GABA synthesis enzyme, Gad65 Glp-1r-/- and Glp-1r+/+ dLS neurons did not differ in passive membrane properties, AP waveform, the minimum current required to start AP or fast AHP amplitude Start of an AP was delayed in Glp-1r-/- dLS neurons compared to Glp-1r+/+ Above-threshold current injections caused Glp-1r-/- dLS neurons to fire more APs than controls Glp-1r-/- mice showed more anxiety behavior on the EPM compared to Glp-1+/+ regardless of AAV treatment Glp-1r-/- mice had an increased acute response to cocaine while AAV-GLP-1R-GFP treated mice did not Glp-1+/+ and Glp-1r-/- mice, but not those treated with AAV, showed sensitization after a cocaine challenge The groups did not differ in baseline locomotion, the change of the difference between days 5-1 and 12-11 Glp-1r-/- mice expressed increased CPP independent of locomotor activity and AAV reversed this effect Glp-1r-/- mice treated with AAV-GLP-1R-GFP had fourfold the GLP-1 in their dLS compared to Glp-1+/+ |
| (Sørensen et al., 2015) | Male NMRI mice (28 - 35 g) for microdialysis and locomotor experiments Male NMRI mice (20 - 22 g) for acute self-administration  Male C57Bl/6 mice (20 - 24 g) for chronic self-administration | Ex-4 (0.3 - 30 μg/kg) IP 90-100 min before cocaine for locomotor activity Ex-4 (10, 30, 100 μg/kg) IP 90 min before self-administration | Exp1: 1, Saline 2. Ex-4 (0.3-30 μg/kg) 3. Saline + cocaine (30 mg/kg) s.c. 4. Ex-4 (0.3-30 μg/kg) + cocaine (30 mg/kg) s.c. Exp2: 1, Saline 2. Ex-4 (30 μg/kg) 3. Saline + D1R agonist, SKF-82,958 (0.01, 0.1, and 1 mg/kg) s.c. 4. Ex-4 (30 μg/kg) + SKF-82,958 s.c. | Nose-poking behavior Locomotor activity FR1 responding for acute and chronic cocaine self-administration/ISH for c-Fos in the striatum High-performance liquid chromatography with electrochemical detection for DA | Ex-4 attenuated basal, cocaine and D1 receptor agonist-induced increased locomotor activity Ex-4 decreased acute self-administration of cocaine without affecting basal response Ex-4 decreased chronic self-administration of cocaine and abolished aversion to the inactive hole Ex-4 entirely abolished the cocaine-induced c-fos expression in the striatum Ex-4 attenuated cocaine-induced dopamine release in the striatum |
| (Sirohi et al., 2016) | GLP-1R KD Nestin mice  FLOX mice | GLP-1R KD Nestin (GLP-1R selectively abolished from CNS) Ex-4 (30 μg/kg) IP | 1. Saline 2. D-amphetamine (1.0 mg/kg, IP) + saline 3. D-amphetamine (1.0 mg/kg, IP) + Ex-4 | Amphetamine-induced CPP Locomotor activity | Ex-4 blocked amphetamine-induced CPP expression in FLOX mice; there was no blockage in GLP-1R KD Nestin mice Locomotor activity did not change in both groups after Ex-4 |
| (Bornebusch et al., 2019) | Male Glp1R flox/flox nestin-Cre/ KO  WT mice | Neuronal-specific deletion of GLP-1R Ex-4 (10 μg/kg) IP 30 min before testing Ex-4 (3.2, 10 μg/kg) IP for ethanol tests 30 min before testing | Exp1-2: 1. Saline 2. Ex-4  Exp3: 1. Saline + morphine 2. Ex-4 + morphine Exp4-5: 1. WT + saline 2. WT + Ex-4 3. KO + saline 4. KO + Ex-4 Exp6: 1. Saline + naltrexone 2. Saline + saline 3. Morphine + saline | CPP for morphine 30 min after injections PR and FR responding for food, remifentanil, ethanol 30 min after injections Locomotor activity during CPP Nose-poking behavior Hot-plate assay Jumps per 30 min for somatic symptoms of morphine withdrawal | In both saline or Ex-4, significant place preference was observed in morphine-conditioned mice in comparison to the saline  Under the FR 1 schedule of reinforcement, the behaviors of GLP-1R KO and WT mice were not different During an increased response requirement test (FR3, FR5), the KO mice earned more reinforcers than the WT mice Remifentanil self-administration did not differ between saline or Ex-4 treatment in both WT and KO mice Only post-extinction re-baseline showed increased remifentanil intake after Ex-4 in WT (but not in KO) mice Ethanol intake was decreased significantly by both Ex-4 doses Morphine withdrawal and jumping was induced by morphine and naltrexone Ex-4 did not change these somatic symptoms of morphine-dependent groups treated with naltrexone and of non-withdrawal control groups The nociceptive response did not differ between saline or Ex-4 in both WT and KO mice |
| **2. Alcohol** | | | | | |
| **2.a Rats** | | | | | |
| (Shirazi et al., 2013) | Male Wistar rats (250 g) Male NMRI mice (22 g) for CPP | Intermittent-access 20% ethanol two-bottle-choice drinking model/GLP-1 (0.02, 0.1 mg/kg) IP Ex-4 (0.3 μg/kg, 1.0 μg/kg) i.p. Ex-9 (0.1 mg/kg) IP GLP-1 (1.0 μg) and Ex-4 (0.1 μg) unilateral VTA microinjections | Exp1: 1. Vehicle 2. GLP-1 3. Ex-4  4. Ex-9  Exp2: 1. Vehicle 2. GLP-1 (0.02 mg) Exp3: 1. Vehicle 2. Intra-VTA GLP-1 3. Intra-VTA Ex-4 | CPP Alcohol consumption at 1 and 24 h 30 min after injections Food and water intake overnight | GLP-1 and Ex-4 decreased 1 h alcohol intake in rats Only in the high drinking group, but not in low drinking group, GLP-1 decreased alcohol consumption without affecting chow or water intake GLP-1 reduced preference for alcohol of mice in CPP test Ex-9 increased alcohol consumption at both 1 and 24 h Intra-VTA GLP-1 reduced alcohol consumption without affecting water and food intake Intra-VTA Ex-4 reduced both alcohol and water consumption Intra-VTA Ex-4 did not change 1h but reduced 16 h food intake |
| (Vallöf et al., 2016) | Mice Outbread Wistar rats Selectively bred alcohol-preferring rats (for operant responding) | In the intermittent access model, rats were offered 20% alcohol 3 days a week for 12 weeks prior to the experiment Rats consuming more than 2.5 g/kg were considered "high-consuming", while others were "low-consuming" Alcohol deprivation model: intermittent access model for 10 weeks, following deprivation for 10 days and reintroduction/Liraglutide (0.05, 0.1 mg/kg) s.c. acute and chronic (for 7 days) | Exp1: 1. Alcohol (time 0) + vehicle (time 140 min) + alcohol (time 200 min) 2. Alcohol + liraglutide + alcohol 3. Vehicle + liraglutide + vehicle Exp2: 1. Vehicle + alcohol 2. Liraglutide + alcohol | CPP Food, water, and alcohol intake 60 min after injection (acute) and test day 1,2 and 3 (chronic) Body weight Intermittent access drinking model Operant responding for alcohol at the end of chronic treatment/In vivo microdialysis for accumbal dopamine Blood alcohol levels | Liraglutide attenuated the alcohol-induced increase in accumbal dopamine  Liraglutide, when applied during, but not post, conditioning decreased alcohol-induced CPP After 12 weeks of alcohol consumption, acute liraglutide decreased food and alcohol intake and alcohol preference and caused a trend towards increased water intake After 12 weeks of alcohol consumption, liraglutide decreased alcohol and food intake high-alcohol-consuming rats but not low-alcohol-consuming rats After 10 weeks of alcohol consumption, liraglutide decreased deprivation-induced drinking and alcohol intake Repeated liraglutide decreased alcohol intake and preference and food intake on day 1-2 but not 3 Repeated liraglutide decreased operant responding for alcohol, food intake, and body weight in selectively bred alcohol-preferring rats |
| (Abtahi et al., 2018) | Adult female Sprague-Dawley rats (225-250 g) | Two-bottle intermittent access paradigm to habituate rats to alcohol for 12 weeks/Intra-NA core and shell Ex-4 (0.025, 0.05 μg) at the onset of the nocturnal cycle | 1. Vehicle + vehicle 2. Ghrelin antagonist, JMV2959 (2 μg) + vehicle 3. JMV 2959 (10 μg) + vehicle 4. Vehicle + Ex-4 (0.025 μg) 5. Vehicle + Ex-4 (0.05 μg) 6. JMV2959 (2 μg) + Ex-4 (0.025 μg) | Two-bottle intermittent choice paradigm Food and water intake (daily, in experiment days 2, 6 and 24 h post-injections)/Vaginal cytology for estrous cycle assessment | JMV2959 decreased alcohol intake when administered into both NAc core (at times 6 ad 24 h) and shell (at all time points) Ex-4 alone or Ex-4 with JMV2959 reduced alcohol intake in the NAc shell but not in the core Ex-4 alone decreased food intake in both NA core and shell time-dependently Ex-4 with JMV2959 reduced food intake in the NAc core, but not in the NAc shell Water (and total fluid) intake decreased during the combined proestrus and estrus phases of the estrous cycle Alcohol or food intake did not experience estrous-related differences |
| (Vallöf et al., 2020) | Adult male and female Rcc Han Wister rats (150-200 g for females, 200-240 g for males) | Intermittent access 20% ethanol two-bottle-choice drinking paradigm/Dulaglutide (0.05 or 0.1 mg/kg) s.c. once weekly for 9 or 5 weeks | 1. Vehicle 2. Dulaglutide | Ethanol, water, total fluid intake/HPLC analysis | Both doses of dulaglutide decreased ethanol intake and ethanol preference after 9 and 5 weeks in both male and female rats Dulaglutide did not significantly change water and total fluid intake After the end of treatment period, reduced ethanol intake was continued but only in male rats After 9 weeks of treatment, dulaglutide changed levels of dopamine, 5-HIAA, serotonin, noradrenaline in the amygdala of male rats and levels of DOPAC, dopamine, noradrenaline in the striatum of female rats |
| (Dixon et al., 2020) | Male Long-Evans rats | Intermittent access 20% alcohol two-bottle-choice drinking paradigm, trained for nose poke for 20% alcohol/Ex-4 (0.01 or 0.5 μg/kg) intra-VTA | 1. Vehicle 2. Intra-VTA Ex-4 | Alcohol self-administration Reacquisition of alcohol self-administration PR schedule of reinforcement Locomotor activity | Intra-VTA Ex-4 at both doses significantly decreased alcohol self-administration (active nose pokes and alcohol deliveries) Intra-VTA Ex-4 did not change extinction following reacquisition of alcohol self-administration nor motivation for alcohol Only the higher dose of intra-VTA Ex-4 significantly reduced food intake Intra-VTA Ex-4 did not change locomotor activity |
| **2.b Mice** | | | | | |
| (Egecioglu et al., 2013c) | Adult post-pubertal male NMRI mice (25 - 40 g) Adult male Rcc Han Wistar rats | Intermittent-access 20% ethanol two-bottle-choice drinking model  Before the intermittent access model experiment, rats consumed alcohol for 8 months Before the PR experiment, rats consumed alcohol for 9 months/Ex-4 (0.3 and 1.2 μg/kg) i.p. | Exp1: 1. Vehicle  2. Alcohol 3. Ex-4 + vehicle  4. Ex-4 + alcohol  Exp2: 1. Alcohol + vehicle + alcohol  2. Alcohol + Ex-4 + alcohol  3. Alcohol + vehicle + vehicle  4. Vehicle + Ex-4 + vehicle Exp3: 1. Ex-4  2. Vehicle | Locomotor activity 5 min after the last injection CPP PR testing 10 min after Ex-4 Alcohol, water intake/In vivo microdialysis for accumbal dopamine every 20 min | Ex-4 increased locomotor activity at 4.8 μg/kg but did not affect 1.2 and 2.4 μg/kg Ex-4 at doses subthreshold, when applied alone, reversed the alcohol-induced increase in locomotor activity in mice Alcohol increased accumbal dopamine release while Ex-4 pretreatment attenuated this effect Ex-4 alone did not affect accumbal dopamine release Ex-4 attenuated alcohol-induced CPP in mice when applied post-conditioning or during conditioning After 8 months of alcohol intake in rats, Ex-4 at both doses decreased 24 h alcohol intake and alcohol preference over water After 8 months of alcohol intake, Ex-4 (1.2, but not 0.3 μg/kg) decreased 1 and 4 h alcohol intake and alcohol preference over water Ex-4 did not cause an alcohol re-bound post-treatment After 9 months of alcohol intake in rats, Ex-4 decreased active lever-pressing for alcohol and inactive presses also, along with reducing breakpoint |
| (Suchankova et al., 2015) | Adult male C57BL/6 mice | Alcohol dependence mouse model/AC3174, an exenatide analog (0.03, 0.10, 0.30 μg/kg) | 1. Control 2. EtOH mice | Voluntary ethanol consumption | AC3174 significantly decreased ethanol intake in alcohol dependent mice |
| (Sirohi et al., 2016) | GLP-1R KD Nestin mice  FLOX mice | GLP-1R KD Nestin (GLP-1R selectively abolished from CNS) Ex-4 (30 μg/kg) IP | 1. 10 % alcohol solution + saline 2. 10 % alcohol solution + Ex-4 3. Water + saline 4. Water + Ex-4 | Alcohol intake | Ex4 blocked alcohol intake in FLOX mice, no difference was observed in GLP-1R KD Nestin mice |
| (Sørensen et al., 2016) | Naive, free-fed 8 weeks old male C57BL/6J mice | 75 or 125 mg/kg/infusion intravenous EtOH solution in a FR for daily 2-hour sessions, 5 to 6 days per week/Ex-4 (3.2 μg/kg and 1.8 μg/kg) IP | 1. Saline vehicle  2. Ex-4 | Operant responding for palatable liquid food intake  Ethanol intake before and after treatment Cumulative ethanol reinforcers earned before and after treatment | Ex-4 (3.2 μg/kg) reduced the EtOH intravenous self-administration, whereas saline injection did not cause any changes Ex-4 (3.2 μg/kg) produced an extinction-like behavior in cumulative ethanol reinforcers earned Ex-4 did not significantly affect palatable liquid food intake |
| (Thomsen et al., 2017) | 24 male C57BL/6NTac mice (10 weeks of age) | Mice consumed alcohol 2% for 9 days, 4% for 7 days, and 8% for 21 days Treatments were conducted on the following 10-day deprivation period, then 8% alcohol was reintroduced/Ex-4 (1.5 μg/kg/day) s.c. for 8 days during alcohol deprivation and 8 days during reintroduction | 1. Saline 2. Ex-4 | Alcohol, water, and food intake daily after the beginning of alcohol deprivation Meal patterns over 8 days for brevity Body weight throughout the experiment | Ex-4 blocked the deprivation-induced increase in drinking One day after Ex-4 was stopped, the mice consumed the same level as saline-treated mice Ex-4 decreased the number of bouts for alcohol but did not affect time spent or the amount consumed per bout Ex-4 treated mice took longer to start consuming alcohol after the deprivation period Ex-4 group drank larger volumes of water per bout but the number of bouts or time spent per bout did not differ Total fluid intake did not differ between treatment or alcohol deprivation Locomotor activity decreased over time but did not depend on treatment |
| (Vallöf et al., 2019a) | Adult post-pubertal male NMRI mice (8-12 weeks old, 25-35 g) Adult post-pubertal male outbred Rcc Han Wistar rats | Intermittent-access 20% ethanol two-bottle-choice drinking model for 12 weeks Rats consuming more than 3.5 g/kg/24h were considered "high-consuming" while others were "low-consuming"/Ex-4 (0.0025, 0.025 or 0.05 μg per side) into NAc shell, anterior and posterior VTA, laterodorsal tegmental area (LDTg) 10 min before testing or alcohol exposure | Exp1: 1. Vehicle + vehicle 2. Vehicle + alcohol 3. Ex-4 + vehicle 4. Ex-4 + alcohol (1.75 g/kg, i.p.) Exp2: 1. Vehicle 2. Ex-4 | Alcohol intake  CPP Locomotor activity /GLP-1R and preproglucagon gene (GCG) levels | Ex-4 into the NAc shell blocked the locomotor stimulation caused by alcohol Intra-NAc shell Ex-4 blocked the alcohol-induced memory retrieval of alcohol reward in CPP Ex-4 into NAc shell reduced alcohol intake, but it did not reduce water intake or body weight Glp-1R expression in NAc shell increased significantly in high consuming mice Glp-1R expression did not differ between high and low alcohol-consuming mice in the PFC, VTA, amygdala, hippocampus, and striatum Intra-pVTA Ex-4 reduced locomotor activity caused by alcohol but did not change CPP or alcohol intake Intra-VTA Ex-4 had no significant effect on locomotor activity and CPP Intra-LDTg Ex-4 blocked the locomotor stimulation caused by alcohol and reduced alcohol intake, but it did not affect CPP |
| (Vallöf et al., 2019b) | Adult post-pubertal male NMRI mice (8-12 weeks old, 25-35 g) Adult post-pubertal male outbred Rcc Han Wistar rats | Intermittent-access 20% ethanol two-bottle-choice drinking model for 12 weeks/Ex-4 (0.05, 0.025 μg per side) into the NTS  Ex-4 (2.4 μg/kg) IP 10 min before alcohol exposure Ex-9 (5 μg per side) into NTS 15 min before Ex-4 | Exp1-2: 1. Ex-4  2. Vehicle  Ex3: 1. Ex-9 + Ex-4 + alcohol (1.75 g/kg, i.p.) 2. Vehicle + Ex-4 + alcohol  3. Ex-9 + vehicle + vehicle 4. Vehicle + vehicle + vehicle | Alcohol, water, total fluid, and food intake at 1, 4, and 24 h after bottle presentation Locomotor activity CPP/Invivo microdialysis measurements Dopamine release measurements | Ex-4 (0.05 μg per side) into the NTS decreased acute effects of alcohol such as locomotor behavior induced by alcohol, dopamine release from the NAc, and reward-dependent memory consolidation in CPP Alcohol intake was reduced at all time points, alcohol preference was decreased at 4 and 24 h with Ex-4 into the NTS in comparison to the vehicle in rats consuming alcohol for 12 weeks/ however, water intake did not change A lower dose of Ex-4 (0.025 μg per side) did not change alcohol intake Ex-9 into the NTS blocked this reducing effect on locomotor activity of Ex-4 |
| (Bornebusch et al., 2019) | Male Glp1R flox/flox nestin-Cre/ KO  WT mice | Neuronal-specific deletion of GLP-1R Ex-4 (10 μg/kg) IP 30 min before testing Ex-4 (3.2, 10 μg/kg) IP for ethanol tests 30 min before testing | Exp1-2: 1. Saline 2. Ex-4  Exp3: 1. Saline + morphine 2. Ex-4 + morphine Exp4-5: 1. WT + saline 2. WT + Ex-4 3. KO + saline 4. KO + Ex-4 Exp6: 1. Saline + naltrexone 2. Saline + saline 3. Morphine + saline | CPP for morphine 30 min after injections PR and FR responding for food, remifentanil, ethanol 30 min after injections Locomotor activity during CPP Nose-poking behavior Hot-plate assay Jumps per 30 min for somatic symptoms of morphine withdrawal | In both saline or Ex-4, significant place preference was observed in morphine-conditioned mice in comparison to the saline  Under the FR 1 schedule of reinforcement, the behaviors of GLP-1R KO and WT mice were not different During an increased response requirement test (FR3, FR5), the KO mice earned more reinforcers than the WT mice Remifentanil self-administration did not differ between saline or Ex-4 treatment in both WT and KO mice Only post-extinction re-baseline showed increased remifentanil intake after Ex-4 in WT (but not in KO) mice Ethanol intake was decreased significantly by both Ex-4 doses Morphine withdrawal and jumping was induced by morphine and naltrexone Ex-4 did not change these somatic symptoms of morphine-dependent groups treated with naltrexone and of non-withdrawal control groups The nociceptive response did not differ between saline or Ex-4 in both WT and KO mice |
| **2.c Monkeys** | | | | | |
| (Thomsen et al., 2019) | 32 drug and experimentally naive young adult male African vervet monkeys (4.6-5.8 kg) | Placebo-controlled studies One bottle containing water and one bottle containing an alcohol solution of 10% was given to the animals Exclusion criteria for alcohol-preferring monkeys: drinking less than 1 g/kg of alcohol in the 4 h period; Exenatide (0.04 mg/kg) weekly for 5 weeks Liraglutide (10 to 50 μg/kg/day, increasing doses) for 2 weeks | Exp1: 1. Vehicle  2. Exenatide Exp2: 1. Vehicle 2. Liraglutide | Alcohol and water intake for daily 4-h drinking sessions at baseline, when drug deliveries were complete and 1 week during washout/Plasma alcohol levels immediately after a 4-h drinking session | During the baseline period in both experiments, there was no difference in alcohol and water intake between vehicle and treatment groups During the treatment period, when alcohol was made available again, liraglutide and exenatide (to a lesser extent) group drank less alcohol than the vehicle group Water intake did not differ between groups during the treatment period In the liraglutide group, alcohol plasma concentrations in blood were significantly lower than the vehicle group, whereas there was no significant difference in the exenatide experiment |
| **3. Nicotine** | | | | | |
| (Egecioglu et al., 2013a) | Adult post-pubertal age-matched male NMRI mice (8-12 weeks old, 25-40 gr) | Ex-4 (2.4 μg/kg) i.p. | 1. Vehicle + vehicle 2. Ex-4 + vehicle  3. Vehicle + nicotine (0.5 mg/kg, i.p.) 4. Ex-4 + nicotine (10 min after) | CPP 10 min after Ex-4 Locomotor activity 15 min after nicotine Locomotor sensitization after 5 subsequent days of nicotine injections and immediately after Ex-4/Invivo microdialysis and dopamine release measurements | Locomotor activity was increased by nicotine compared to the vehicle; however, Ex-4 significantly reduced nicotine-induced locomotor activity Accumbal dopamine release was increased by nicotine compared to vehicle and Ex-4 significantly reduced this release  Ex-4 attenuated the nicotine-induced expression of CPP  Subchronic nicotine (for 5 days) resulted in locomotor sensitization and a single injection of Ex-4 abolished this sensitization |
| (Tuesta et al., 2017) | DREADD: 6-week old Gcg-Cre and Phox2b-Cre mice Gcg-Cre crossed with ROSA-tdTom mice for NTS GLP-1 detection: ChAT-ChR2-YFP mice for electrophysiological recordings Chrna5-EGFP reporter mice for NTS measurements Male Wistar rats (275–300 g) | GLP-1R KO Ex-4 (10 μg/kg) Ex-9 (20 μg) Sitagliptin  Chemogenetic GLP-1 activation | Exp1: 1. Saline 2. Nicotine (0.25-1.5 mg/kg) Exp2: 1.Vehicle 2. GLP-1 KO  3. Ex-4 4. Sitagliptin | Operant responding for nicotine 1-h operant responding for food reward/IHC for GLP-1, GFP, TH and Fos/Whole-cell voltage-clamp recordings | Nicotine activated GLP-1 neurons, but not TH+ neurons, in NTS Nicotine activated TH+ neurons in the ventrolateral medulla GLP-1 neurons expressed α5 nAChR Ex-4 and sitagliptin decreased nicotine intake, but not food rewards Glp1R KO mice ingested more nicotine compared to WT but did not differ in food responses GLP-1 was found to be not involved in the ability of nicotine to suppress appetite Chemogenetic stimulation of NTS GLP-1 neurons decreased nicotine intake NTS GLP-1 neurons were found to activate the interpeduncular nucleus (IPN) neurons through habenular terminals to decrease nicotine intake Intra-IPN GLP-1 decreased nicotine intake but did not change food reward and cAMP inhibitors reversed this effect Intra-IPN Ex-9 increased nicotine intake (seen 24 h after infusion) but did not change food reward GLP-1 in the MHb-IPN circuit worked to reduce the amount of nicotine consumed without causing malaise |
