# Supplementary material for: Can GLP-1 Be a Target for Reward System Related Disorders? A Qualitative Synthesis and Systematic Review Analysis of Studies on Palatable Food, Drugs of Abuse, and Alcohol
Source: Front Behav Neurosci. 2021 Jan 18;14:614884. doi: 10.3389/fnbeh.2020.614884 (PMC7848227; doi:10.3389/fnbeh.2020.614884)
Supplement: Supplementary file 3 [file Table_3.DOCX]

| Supplementary Table 3. Studies on humans about GLP-1 and reward | | | | | |
| --- | --- | --- | --- | --- | --- |
| ***Name of First Author/ Publication Year*** | ***Participants*** | ***Design of the Study/Application of GLP-1 agonist (dose and method)*** | ***Experimental Groups*** | ***Assessments*** | ***Main Findings*** |
| **1. Food intake** | | | | | |
| (Pannacciulli et al., 2007) | 42 Caucasian subjects (22 men and 20 women, age 31 years, body fat 7-44%) | Weight maintaining diet (50% carbohydrate, 30% fat, 20% protein) | 1. Fasted state 2. Postprandial state | Resting energy expenditure (REE) measurements after an overnight fast for 45 min after each scan Dual-energy X-ray absorptiometry for body composition/Plasma glucose, insulin, and serum FFA levels  Total plasma GLP-1 levels  Blood samples were taken after each scan/PET measured regional cerebral blood flow (rCBF) after 36 h of fasting MRI 2 sessions at baseline and after oral administration of a satiating amount of liquid meal | There was a significant positive correlation between postprandial changes in plasma GLP-1 levels and left dLPFC (including left middle and inferior frontal gyri) and hypothalamus rCBF These changes were not related to glucose, insulin and FFA levels Between post-meal changes in plasma GLP-1 concentrations and rCBF in the dLPFC, significant negative associations were not observed |
| (De Silva et al., 2011) | 16 (11 males, 5 females) healthy humans (BMI: 22.1) | Single-blinded randomized study consisting of 5 visits Measurements after an overnight fast/GLP-17-36 amide infusion (0.8 pmol/kg/min) for 90 min | Over five visits, after an overnight fast: 1. Fasted saline (saline only for 90 min) 2. Fed saline (2500 KJ breakfast + saline for 90 min 3. PYY3-36 (90 min, 0.3 pmol/kg/min) 4. GLP-17-36 amide  5. PYY3-36 + GLP-17-36 amide | VAS ratings of food and nonfood-related sensations at -60, 0, 90, 120 min Food intake at open buffet immediately after scanning/Plasma PYY and GLP-1 levels  Blood glucose levels/BOLD fMRI for 60 min after 25 min after infusion ROI: insula, caudate, amygdala, OFC, putamen, NAc | GLP-1 and PYY, both separately and combined, along with breakfast decreased energy intake on the following lunch Breakfast and combined GLP-1+PYY decreased reported hunger in VAS After saline, ROI activity increased from viewing food to non-food pictures regardless of fullness In fasted GLP-1+PYY and PYY rats, ROI activity decreased from food to non-food pictures when compared to fasted saline In fasted GLP-1 rats, ROI activity did not differ between food and non-food pictures when compared to fasted saline In fed GLP-1, PYY, or both infused rats ROI activity decreased from food to non-food pictures compared to fasted saline Reduction in left NAc and left OFC activity for fed PYY group was significant versus fasted saline Reduction in right insula activity for fed GLP-1 group was significant versus fasted saline |
| (Lemmens et al., 2011) | 18 men and 20 women (mean age: 24, BMI: 25.0) | Randomized crossover design 2 visits (at least 1 week apart in a fasted state) for a 4-course lunch in 0.5 h (non-staggered) or in 2 h with 3 within-meal pauses (staggered) followed by ad libidum food intake | 1. Non-staggered 2. Staggered | Body weight, height, waist circumference, and hip circumference measurements Energy intake Ad libidum consumption of sweet and savory food items Computer test for "liking" and "wanting" before and after 4-course lunch and after ad libidum buffet Visual analog scale (VAS) on appetite before and during testing/GLP-1, PYY3–36, and ghrelin plasma levels throughout the test sessions | Peak values of GLP-1, PYY3–36, and satiety were lower in the staggered than non-staggered meal condition Before ad libitum food intake, GLP-1 concentrations and ratings for satiety were higher, whereas, concentrations of ghrelin, hunger ratings, desire for food ("wanting") was less in the staggered compared to non-staggered meal condition, but this situation did not alter ad libitum energy intake  Food "wanting" after meal intake did not correlate with GLP-1, ghrelin levels and satiety, hunger ratings in both staggered and non-staggered condition |
| (van Bloemendaal et al., 2014) | 16 obese T2DM patients 16 obese normoglycemic 16 healthy lean individual | Randomized, crossover, placebo-controlled trial 3 days prior to the examination, sulfonylureas was discontinued, whereas metformin was discontinued on the day of examination Somatostatin pancreatic-pituitary clamp to suppress endogenous GLP-1 production/Exenatide (50 ng/min for 30 min and was decreased to 25 ng/min) Ex-9 | 1. Lean + Control 2. Obese + Control 3. T2DM patient + Control 4. Lean + Exenatide 5. Obese + Exenatide 6. T2DM patient + Exenatide 7. Lean + Exenatide + Ex-9 8. Obese + Exenatide + Ex-9 9. T2DM patient + Exenatide + Ex-9 | Hunger, fullness, appetite, prospective food consumption, desire to eat  Profile of mood states (POMS) before and after fMRI scan and ad libidum lunch Food intake before and after fMRI/HbA1c, total cholesterol, LDL, HDL, triglycerides levels Fasting insulin, fasting glucagon, fasting growth hormone, fasting cortisol levels/fMRI for food picture evaluation for 3 visits at least 1 week apart  ROI: insula, striatum, amygdala, OFC | Right insula of obese subjects was more active (food vs non-food, high-calorie vs non-food) than that of T2DM in response to food pictures Exenatide decreased activations in bilateral insula, left putamen, and right OFC (food vs non-food, high-calorie vs non-food) of T2DM Exenatide decreased activations in the right insula and left OFC (high-calorie vs non-food) of obese subjects Caloric intake was significantly reduced in exenatide versus saline injections and these effects were decreased by concomitant infusion with Ex-9 There was a positive correlation between caloric intake of exenatide vs placebo and CNS activations for food vs non-food and exenatide vs placebo in the bilateral insula and right caudate nucleus in T2DM and bilateral OFC, bilateral insula and right caudate nucleus in obese subjects |
| (Jennifer et al., 2015) | 20 overweight and obese patients with T2DM  20 healthy lean individuals (normoglycemic) | Randomized, crossover, placebo-controlled intervention study 2 separate visits of 2 fMRI scan each Intake of a standardized liquid meal (carbohydrate 56.1 g, fat 17.4 g and protein 18.0 g, 300 ml Nutri-drink yogurt style)/Ex-9 (600 pmol/kg/min, i.v.) injected 1 h before and continued during the whole scanning session | Exp1: 1. Placebo 2. Ex-9 Exp2: 1. Healthy controls 2. T2DM obese patients Exp3: 1. Fasted state 2. Postprandial state | 10-point Likert scale before the start of first MRI (fasted), before intake of the meal, 30 and 60 min after intake/Blood glucose, insulin, glucagon levels before the start of first MRI (fasted), before intake of the meal, 30 and 60 min after intake/fMRI during the evaluation of food pictures, fasted and postprandial state (during the meal and 30 minutes after intake of the standardized liquid meal) 2 sessions for Ex-9 and saline | In the fasting condition, CNS activation in bilateral insula, left amygdala and right OFC was increased by food pictures and in the right OFC and left insula by high-energy food pictures in T2DM patients compared to healthy lean controls In the postprandial condition, CNS activations of the regions mentioned above were no longer observed CNS activation was decreased after meal intake in the bilateral insula by food pictures and in the left insula, left caudate nucleus, and right OFC by high-energy food pictures in obese T2DM patients In healthy lean controls, meal intake reduced CNS activation in the right insula in response to food and high-energy food pictures but not as significant as it was in T2DM patients This reducing effect was demolished with the blockage of GLP-1 in bilateral insula to food pictures in obese T2DM patients 60 minutes after the meal, the hunger scores of Ex-9 injected T2DM was higher than the placebo injected T2DM patients; no differences were observed in healthy lean controls |
| (van Bloemendaal et al., 2015a) | 16 obese with T2DM (BMI >30 kg/m2) 16 obese with normoglycemia (BMI >30 kg/m2) 16 healthy lean (BMI <25 kg/m2) | A randomized, placebo-controlled, crossover study All tests were performed during a somatostatin pancreatic-pituitary clamp to eliminate cofounders/Exenatide (50 ng/min for 30 min and decreased to 25 ng/min, intravenous) Ex-9 (600 pmol/kg/min) | 1. Exenatide 2. Exenatide + Ex-9 3. Saline | 10-point Likert scale start and 50 min of the clamp, before fMRI task, before and after ad libidum lunch Choices and food intake assessed by ad libidum lunch buffet/Chocolate milk fMRI paradigm (3 sessions on separate visits, with at least 1 week between sessions) | BMI positively correlated with brain responses in the bilateral caudate nucleus, bilateral putamen, and right insula to the anticipation of receipt of palatable food and negatively correlated with brain responses in the right putamen to the actual receipt of the palatable food in the placebo condition Exenatide increased brain activations in consummatory food reward in the right caudate nucleus of lean subjects, in right OFC of obese subjects and left insula, bilateral putamen, and left amygdala of obese with T2DM and Ex-9 attenuated these activations Exenatide decreased anticipatory food reward in bilateral OFC of lean subjects, in bilateral putamen, left insula, and left amygdala of obese with T2DM and Ex-9 attenuated these activations |
| (van Bloemendaal et al., 2015b) | 16 obese T2DM patients 16 obese normoglycemic 16 healthy lean individual (40-70 years) | Randomized, placebo-controlled crossover study Somatostatin pancreatic-pituitary clamps Overnight fast/Exenatide (50 ng/min for 30 minutes, and after 25 ng/min, intravenous) | 1. Lean + Placebo 2. Obese + Placebo 3. T2DM obese + Placebo 4. Lean + Exenatide 5. Obese + Exenatide 6. T2DM obese + Exenatide | Recognition test DEBQ-Emotional Eating subscale Ad libidum lunch buffet to evaluate energy intake after the fMRI/fMRI paradigm for food picture evaluation on 2 visits | A positive correlation was observed in axial brain slices in subjects with obesity in right insula between emotional eating and brain responses to food versus non-food pictures In the amygdala, a negative correlation was observed between emotional eating and exenatide-induced reductions in brain responses to food versus non-food pictures in obese patients, however, this negative correlation was insignificant when adjusted for BMI In left and right amygdala, inferior OFC, and right insula, there was a positive correlation in T2DM patients with obesity between emotional eating and brain responses to food versus non-food pictures In the right insula, a negative correlation was observed between emotional eating and exenatide-induced reductions in brain responses to food versus non-food pictures in obese T2DM patients |
| (Heni et al., 2015) | 12 lean individuals 12 obese individuals | 75g oral glucose test to promote GLP-1 secretion | 1. Lean 2. Overweight/ obese | Plasma insulin and glucose levels Plasma GLP-1 levels (before, 30, and 120 min after glucose ingestion)/fMRI on whole-brain to food cues before, 30, and 120 min after glucose intake | GLP-1 changes negatively correlated with food cue-induced OFC activity from before to 120 min after glucose intake in both lean and obese individuals This was independent of the changes in insulin and glucose concentrations, gender, BMI, and age Only in lean individuals, postprandial insulin changes were associated with OFC activations |
| (Daniele et al., 2015) | 15 pre-diabetic human males (age: 57 years, BMI: 29.4) | Randomized, double-blind, placebo-controlled trial Pre-diabetes with HbA1c: 5.7, fasting glucose: 114 mg/dl and 2h-glucose: 177 mg/dl 75 g glucose drink in 5 minutes /Exenatide (5 μg) s.c. | 1. Exenatide 2. Placebo | Oral glucose test (OGTT) 30 min after injection/Plasma F-FDG radioactivity and glucose concentrations every 15 min Plasma insulin and tracer enrichments every 15 min Urine volume, FDG radioactivity, and glucose concentrations from urine samples at the end of OGTT/2 PET scans for both conditions within 26 days 2D dynamic brain scan, 10 min  ROI: cortical and subcortical GM regions of brain, glucose homeostasis regulation areas as NTS, brain stem, insula, putamen, caudate, amygdala, limbic system, hypothalamus, and food reward system as OFC, thalamus, anterior and posterior cingulate | Exenatide did not affect the fasting plasma glucose or insulin levels Plasma glucose concentration was close to fasting level with exenatide Plasma insulin level increased twice as much in placebo compared to exenatide Exenatide increased cerebral glucose metabolic rate (CMRglu) in all areas of the brain involved in glucose homeostasis regulation and food reward system compared to placebo; however, exenatide decreased CMRglu for the hypothalamus Exenatide decreased the rate of glucose absorption (RaO) RaO and RaT negatively correlated only with brain areas involved in glucose homeostasis regulation RaO and RaT did not correlate with CMRglu and EGP, clearance (Cglu), or glucose Rd |
| (Rigamonti et al., 2015) | 10 human males (age 33.9 years, BMI 42.6 kg/m2) | 12 hour fast before the test day 1 h after a 300 kcal breakfast (T0=0 min), palatable food presented (T60) for 10 min (during this time they were prohibited from consuming the food), after 10min (T70) participant was allowed to consume the palatable food | 1. Palatable food (milk chocolate tablet) 2. Non-palatable isocaloric food (bread and butter) under the same conditions, 1 week after palatable food consumption | BMI, fat-free mass (FFM), fat mass (FM) Hunger and satiety rating on visual analog scales 3 times (before, during, and immediately after the experiment) Amount of consumed food and calories measured by mass (at the end of the experiment)/Plasma serum ghrelin, PYY, GLP-1 levels Insulin and glucose levels AEA, 2-AG, oleoylethanolamide (OEA), and palmitoylethanolamide (PEA) levels | Higher circulating levels of ghrelin was present for the palatable food session at all time points compared to non-palatable food  Ghrelin secretion was significantly inhibited after breakfast in obese patients in the palatable food session only Experience with palatable food (10 min of exposure without consumption) further increased ghrelin secretion Levels of PYY and GLP-1 was low and did not change before and after exposure and ingestion of palatable and non-palatable foods, only a slow increase in PYY levels was observed after the second part of the two sessions of eating  AEA, 2-AG, and OEA levels increased before the exposure of palatable food Hunger scores were higher and satiety scores were lower in the palatable food compared to non-palatable food session |
| (Jennifer et al., 2016) | 20 obese T2DM patients (40-65 years old) Stable body weight (<5% change in last 3 months), BMI> 26 kg/m2 HbA1c level 42-69 mmol/mol (6.0–8.5%) | All patients were treated with metformin for T2DM Crossover intervention study of two treatment periods with a 12 week washout period in between/Liraglutide (1.8 mg) for 12 weeks (0.6-1.8 mg, with weekly increments of 0.6 mg) | 1. Liraglutide  2. Active comparator, insulin glargine (initial 10 lU q.d., increase in daily dose based on their self-monitored blood glucose levels) | Energy intake assessment (ad libidum lunch buffet 3 h 30 min after intake) Scores of hunger, fullness, prospective food consumption, and nausea on a 10-point Likert scale at five fixed time points in each visit Weight, BMI, waist circumference/Blood glucose levels Blood pressure Heart rate HbA1c, total cholesterol, triglycerides levels/6 fMRI visits after an overnight fast with 2 fMRI scan performed per visit (fasted and 30 min after standardized liquid meal) At the start (baseline), 10 days after (short-term), 12 weeks after (longer-term) 10 min presentation of 1) high-calorie food 2) low-calorie food 3) non-food items | Liraglutide decreased CNS activation in the bilateral insula (in fasted condition) and left putamen (post-prandial condition) in response to viewing of food/high-calorie food pictures after short-term treatment (10 days), compared to insulin  No differences of CNS activation were observed between groups in the longer-term treatment (12 weeks) Hunger scores were lower in liraglutide both at short and long-term compared to insulin; other appetite-related scores were not significantly different Weight loss after 12 weeks and changes of CNS activation in bilateral insula after liraglutide were positively correlated (however, failed to reach significance) CNS activation in significant areas did not correlate with food intake at ad libidum lunch buffet and hunger scores |
| (ten Kulve et al., 2016) | Exp1: 20 healthy lean individuals, 20 obese patients with T2DM Exp2: 20 overweight or obese patients with T2DM | All patients were treated with metformin for T2DM, 12 of the T2DM were treated with extra sulphonylurea 10 patients were treated with antihypertensive medication and 15 patients with cholesterol-lowering agents Exp1: 2 visits, overnight fast before the treatment Exp2: Randomized, cross-over intervention study/Ex-9 (600 pmol/kg/min, i.v.) Liraglutide (dose-escalation period, starting at 0.6 mg once daily, weekly increments of 0.6 mg, final dose of 1.8 mg) | Exp1: 1. Healthy controls  2. Healthy controls + Ex-9 3. Obese patients with T2DM 4. Obese patients with T2DM + Ex-9 Exp2: 1. Insulin (baseline) 2. Liraglutide (baseline) 3. Insulin (10 days) 4. Liraglutide (10 days) 5. Insulin (12 weeks) 6. Liraglutide (12 weeks) | Body composition  Blood pressure Nausea score/HbA1c, fasting plasma glucose, total cholesterol, triglycerides, GLP-1 levels at 0, 30, 60 min/fMRI paradigm of chocolate milk and tasteless solution for 45 min after meal intake | Ex-9 increased the glucose levels of T2DM and increased the glucagon levels of T2DM and lean controls T2DM decreased activation to chocolate milk in the right insula compared to lean controls Ex-9 suppressed activation in the bilateral insula in lean controls Liraglutide caused significant weight loss compared to insulin glargine treatment Liraglutide and insulin glargine decreased glucose levels, but the decrease was higher with liraglutide Activation to chocolate milk in the right insula and caudate nucleus (10 days) was increased by liraglutide/ these effects did not exist after 12 weeks |
| (Goldstone et al., 2016) | 11 RYGB surgery patients after 5 months 9 gastric banding (BAND) patients 10 unoperated non-obese patients | Randomized, placebo-controlled, double-blind, crossover study 10 min after injections, participants consumed 1) ad libidum breakfast of their choice to stimulate gut hormone release and achieve satiety 2) a standardized milkshake breakfast/Acute 1 ml somatostatin analog, octreotide (Sandostatin, 100 μg) to suppress several plasma gut hormones, including GLP-1 | Exp1: 1. Control + saline 2. Control + octreotide 3. RYGB + saline 4. RYGB + octreotide Exp2: 1. BAND (as controls) + saline 2. BAND (as controls) + octreotide 3. RYGB + saline 4. RYGB + octreotide | Food intake VAS for hunger and appetite ratings 1 h after breakfast Progressive ratio task (PRT) for chocolate sweets for 2 sessions/Serum insulin, plasma glucose, total GLP-1, total PYY and FGF19 levels before and after fMRI scan/Food picture evaluation fMRI paradigm Auditory-visual control fMRI paradigm (to make sure the signal is not nonspecific) | Acute octreotide lowered postprandial plasma satiety gut hormones (GLP-1, PYY, serum insulin, FGF19) after RYGB In controls, only GLP-1 levels were lowered after acute octreotide There was no significant effect of octreotide on glucose levels in neither of the groups Acute octreotide increased the breakpoint (amount of work to obtain reward) for a chocolate sweet in PRT compared to controls/ saline injections did not affect PRT There was no correlation between PRT breakpoint and plasma gut hormones with octreotide Octreotide increased food appeal ratings during the evaluation of food pictures after RYGB but not BAND surgery/ this increase correlated with activation of reward system regions (NAc, caudate, anterior insula, amygdala) PYY and GLP-1 had more significant roles in altering the reward system BOLD signal with octreotide The decrease of PYY and GLP-1 with octreotide was lower in the BAND patients than RYGB patients, associated with higher postprandial concentrations (particularly of GLP-1 after RYGB than BAND in the saline visit |
| (Byrne et al., 2016) | 20 non-obese healthy men (age 18–65 years, BMI: 20–35) | Randomized, placebo-controlled, within-subject, single-blind crossover study 10 g inulin-propionate ester (IPE/ selectively augments colonic propionate production, treatment) or 10 g inulin (control) at a random order | 1.Inulin propionate ester 2.Inulin | Food appeal ratings Food intake/Serum insulin, PYY, and GLP-1 levels were measured with radioimmunoassay/BOLD fMRI of food picture evaluation ROI: caudate, NAc, amygdala, anterior insula, OFC | Decreased activation was seen after increment of colonic propionate production in the NAc and caudate nucleus Response to high energy food was decreased by decreased activation in the caudate Colonic propionate production in striatal brain reward systems decreased ad libitum food intake These observations were not related to changes in blood peptide YY, GLP-1, glucose, or insulin concentrations |
| (Farr et al., 2016a) | Exp1: 13 male 9 female human brains (age: 34-95) less than 24h after death Exp2: 9 male 9 female T2DM humans (age: 48.9 years) | Exp2: Randomised, crossover, placebo-controlled, double-blind study for a total of 17 days/Gradually increasing doses of liraglutide (visit 1: 0.6 mg, visit 2: 1.2 mg, visit 3: 1.8 mg) | 1. Liraglutide  2. Placebo (Conditions separated by min 3 weeks or max 3 months) | VAS for hunger, appetite, and nausea before and after the scan Neurocognitive testing (IED, SSP, SSt, SWM, VRM) after fasting MRI and consumption of a meal/IHC for GLP-1R in the hypothalamus, medulla oblongata, parietal cortex (24 h after death)/fMRI to food cues after an overnight fast of at least 12 h | GLP-1R were abundant in the parietal cortex and was found in the paraventricular nucleus, arcuate nucleus, and ventromedial nucleus In the medulla, the dorsal motor nucleus of the vagus and the area postrema expressed GLP-1 in all cases Liraglutide decreased the energy intake of participants and improved levels of fasting blood glucose but did not affect HbA1c or fructosamine within the time period Neurocognitive testing results did not differ between placebo and liraglutide Participants felt fuller when on liraglutide than placebo Participants’ ratings of hunger and appetite positively correlated with activity in the precuneus, cuneus, parietal cortex, and other occipital cortex areas while using liraglutide Ratings of nausea negatively correlated with activity in the cuneus, precuneus, cingulate cortex, and some parts of the PFC |
| (Farr et al., 2016b) | 20 T2DM patients | Randomized, double-blind, placebo-controlled, cross-over study/Two cross-over phases One phase consisted of 4 visits, the first three one week apart and the last 3 days later Visit 1: 0.6mg liraglutide for one day Visit 2: 1.2 mg liraglutide for one day Visit 3: 1.8 mg liraglutide for 3 days until visit 4 Visit 4: MRI | 1. Liraglutide 2. Placebo | VAS ratings/Fasting serum GIP, amylin, pancreatic polypeptide, fibroblast GF-21, irisin, leptin, GLP-1, ghrelin, PYY, adiponectin, fructosamine, glucose, triglycerides, HDL, LDL, total cholesterol, lipase, and amylase levels at visits 1 and 4 for both phases/fMRI (3-T) fed and fasted while participants rated images after an overnight fast | Participants consumed fewer calories when on liraglutide compared to placebo Percent change of serum leptin was decreased with liraglutide, and it negatively correlated with ratings of fullness GIP and GLP-1 levels were increased with liraglutide, while no other serum parameters were affected Liraglutide did not alter brain activity in response to food cues in the fed state GIP levels inversely correlated with fasting insula activation while viewing highly desirable compared to less desirable foods Changes in leptin positively correlated with activation in the thalamus, pre-SMA, and parietal cortex, and negatively correlated with activation in the dlPFC, midbrain, precuneus, cerebellum, and motor cortex |
| (Jennifer et al., 2017) | 10 female RYGB candidates (age: 40-65 years, BMI > 35) | All patients had laparoscopic RYGB surgery Exclusion criteria: A history of neurological disease, the use of any centrally acting agent, psychiatric disorders, or current diabetes 4 separate test visits: Two of them 8 weeks to 2 weeks before RYGB, and the other two were 4 weeks after RYGB After an overnight fast/Ex-9 (600 pmol/kg/min) started 1 h before and continued during fMRI | 1. Pre-RYGB + placebo 2. Post-RYGB (4 weeks after) + placebo 3. Pre-RYGB + Ex-9 4. Post-RYGB + Ex-9 | Body composition Questionnaires for sensations of hunger, fullness, prospective food consumption, and nausea and appetite for sweet, savory, or fast food items before MRI, before meal, 30 min, and 60 min after meal/Radioimmunoassay for GLP-1 and glucose levels /2 fMRI scans for visual and gustatory (chocolate milk) food cues (one fasted and one 30 min after intake liquid intake) | In fasted condition, CNS activation in response to visual (viewing food pictures) food cues was reduced in the rolandic operculum and caudate nucleus after RYGB Postprandially, CNS activation in response to gustatory (consumption of chocolate milk) food cues were reduced in the insula after RYGB RYGB reduced the effects of GLP-1R blockade as Ex-9 resulted in greater activation in the caudate nucleus to visual food cues and in the insula to gustatory food cues after RYGB compared to before RYGB Feelings of hunger, prospective food consumption, and appetite for sweet and savory food items were reduced after RYGB, along with increased nausea Ex-9 did not change VAS scores on any items GLP-1 levels increased significantly 4 weeks after RYGB |
| (Coveleskie et al., 2017) | 11 lean, 8 obese female subjects | Pre-menopausal and in the follicular phase of the menstrual cycle  After at least 8 hours of fasting  2 day double-blind crossover study/Exenatide (5 μg) s.c. | 1. Lean + Placebo 2. Obese + Placebo 3. Lean + Exenatide 4. Obese + Exenatide | VAS for hunger/satiety level at 25, 35, 60 min after injection Mean meal consumption after scanning/Glucose and exenatide levels at 25, 35, 60 min after injection/fMRI paradigm at fast and 30 min after injection ROI: thalamus, hypothalamus, NTS | In comparison to the placebo, the obese group showed less hunger after exenatide at 35 min and a more significant decrease in hunger scores after meal consumption In the obese group, exenatide resulted in greater functional connectivity between the left hypothalamus and thalamus and left NTS; this situation was not observed in the lean group  In both obese and lean group, exenatide resulted in greater functional connectivity between the left thalamus and right NTS, but this increase was greater in obese Exenatide induced a positive correlation between hunger ratings and functional connectivity between right NTS and right hypothalamus in both lean and obese groups A greater correlation was observed in the lean group between hunger and right NTS functional connectivity with the left thalamus before exenatide administration Correlation between hunger and right NTS functional connectivity with the left thalamus was more significant after exenatide injection in the obese group |
| (Dorton et al., 2018) | 22 lean individuals (BMI 22.6 kg/m2, total body fat 20.6) | Weight stable for 3 months, non-dieters, not on any medication | 1. Ingestion of 75 g of glucose and 0.45 g of non-sweetened cherry flavoring dissolved in 300 ml of water  2. Ingestion 300 ml of water with flavoring was used as a control (Before scanning) | VGA for hunger before and 75 min after drink consumption Multipass 24 h dietary recall (3 to 6 times over 2 months) 24 h physical activity recall (PAR)/Blood sample analysis before and 75 min after drink consumption Luminex multiplex for GLP-1 and PYY levels/fMRI scans: BOLD signals to food vs non-food cues in the bilateral dorsal and ventral striatum after 12 h fast ROI: bilateral dorsal and ventral striatum | As the dietary sugar intake increased, dorsal striatal reactivity to food cues (compared to non-food cues) after glucose consumption increased, even after correcting for sex, percent body fat and average daily physical activity There was a trend of a positive correlation between NAc reactivity to food cues and percent added sugar intake  GLP-1 levels were higher 75 min after glucose consumption compared to baseline, PYY levels were also enhanced but non-significantly Water consumption did not change GLP-1 and PYY levels Higher sugar intake was associated with lower GLP-1 response to glucose consumption, PYY showed no correlation Higher sugar intake did not change GLP-1 and PYY responses to water consumption GLP-1 response to glucose ingestion correlated negatively with dorsal striatum activation to food cues and did not show any correlations with NAc response to food cues Hunger ratings did not correlate with GLP-1 or dorsal striatum response to food cues |
| (Meyer‐Gerspach et al., 2018) | 12 healthy male volunteers (21-31 years) | Randomized, crossover study 2 visits (min 3 days apart) Participants avoided alcohol, exhaustive exercise, and caffeine for 24 hours and to fast overnight for 10 hours before the treatment Intragastric (ig) glucose load (75 g glucose dissolved in 300 mL tap water)/Ex-9 (600 pmol/kg/min, i.v.) | 1. Ex-9 + intragastric glucose (iv-ex9-39/ig-gluc) 2. Intragasic glucose only (ig-gluc) | VAS for hunger, prospective food consumption, satiety and fullness at -10, -1, +15, +60 min Heart rate measurement /Plasma-active GLP-1, GIP, insulin and glucose levels at -10, -1, +15, +60 min/Resting-state functional connectivity (rsFC) analysis by fMRI for 5 min, 10 min after glucose ingestion ROI: hypothalamus, left and right NAc and VTA | After iv-ex9-39/ig-gluc, rsFC was higher between the hypothalamus and left amygdala and hypothalamus and left lateral OFC compared to ig-gluc  After iv-ex9-39/ig-gluc, rsFC was higher between right NAc and the right lateral OFC compared to ig/gluc After iv-ex9-39/ig-gluc, rsFC was lower between the midbrain and the right caudate nucleus After ig/gluc, the sensation of fullness compared to pre-infusion baseline was increased, but prospective food consumption was decreased; these effects were not observed after iv-ex9-39/ig-gluc injection The main effect of treatment and treatment by time interaction effect was not significant for hunger, prospective food consumption, satiety, and fullness After both treatments, plasma GLP-1 concentrations were increased but did not reach significance, although, iv-ex9-39 treatment resulted in higher levels compared to ig-gluc alone |
| (Basolo et al., 2019) | 115 volunteers (72 men, 78 women) | Standard weight-maintaining diet (50% carbohydrates, 30% fat and 20% protein) | 1. Low-fat/high-simple sugar (LF/HSS) 2. Low-fat/high-complex carbohydrate (LF/HCC) 3. Low-fat/high-protein (LF/HP) 4. High-fat/high-complex carbohydrate (HF/HCC)  5. High-fat/high-protein (HF/HP)  6. High-fat/high-simple sugar (HF/HSS) | Ad libidum food intake by vending machine paradigm over 3 days  75g oral glucose tolerance test Body composition analysis/Fasting plasma GLP-1 levels at 1st and 4th days Automated immunoenzymometric for insulin | After 3 days of ad libitum food intake, plasma GLP-1 concentrations increased by 14% On day 1, fasting plasma GLP-1 correlated negatively with carbohydrate intake and with daily energy intake from low fat-high simple sugar |
| (Smits et al., 2019) | Exp1: 16 healthy lean, 16 obese normoglycemic and 16 obese patients with T2DM  Exp2: 20 overweight or obese patient with T2DM | Exp1: Subjects underwent 3 visits in random order with a washout period of more than 1 week Exp2: Randomized, crossover, intervention study for 3 visits (baseline 10 days and 12 weeks after treatment)/Exp1: Exenatide for 90 min Exp2: Liraglutide once daily (< 1.8 mg subcutaneously) for 12 weeks | Exp1: 1. Lean + Placebo 2. Lean + Exenatide 3. Lean + Exenatide + Ex-9 4. Obese + Placebo 5. Obese + Exenatide 6. Obese + Exenatide + Ex-9 7. T2DM + Placebo 8. T2DM + Exenatide 9. T2DM + Exenatide + Ex-9 Exp2: 2.1. Insulin glargine 2.2. Liraglutide | Food questionnaire before and after the scan Ad libitum lunch buffet for the amount of consumed sodium and kcal for 30 min after scanning Salt craving test by a Likert scale at the beginning and before ad libidum lunch/fMRI paradigm during injections and after an overnight fast | Exenatide decreased sodium intake in all groups, but there was not a difference in sodium intake in placebo or exenatide/ex-9 groups Exenatide reduced food intake in all groups |
| (Baboumian et al., 2019) | 55 obese participants (48 females, with BMI:  42.5 and seven males, with BMI:  42.1) | Inclusion criteria: 40.0 < BMI < 55.0 kg/m2, age 18--65 years Roux-en-Y Gastric Bypass (RYGB) (n=16) and sleeve gastrectomy (SG) (n=9) patients Weight loss (WL) program: a 3-month program of cognitive-behavioral therapy, nutritional counseling, nutritionally complete packets | 1. RYGB 2. SG 3. Low-calorie diet weight loss (WL) 4. Non-treatment (NT) | VAS before fMRI scans/Total GLP-1 levels before and after liquid meal/fMRI paradigm for high-energy dense (HED) vs. low-energy-dense (LED) visual and auditory food cues (after a 12 h fast) before and 4 months after surgery | In RYGB, post-surgical increases in dlPFC activation for HED > LED were greatest compared to all the other groups Increased dlPFC activation was seen in SG relative to WL and NT; no significant differences were found between WL and NT As compared to WL and NT, RYGB and SG groups had significantly decreased PHG/fusiform activation for HED > LED GLP-1 postprandial concentrations increased from pre to postsurgical interventions (RYGB, SG), WL intervention or NT did not change it  GLP-1 postprandial concentration increases correlated with post-surgical decreases in the inferior temporal gyrus and right middle occipital gyrus and increases in right medial prefrontal gyrus/paracingulate cortex for HED > LED in RYGB |
| (Maurer et al., 2019) | 156 overweight or obese subjects (120 females, 36 male) | Weight loss-weight maintenance intervention study for 27 months a 12-week weight reduction program, followed by 12-month maintenance phase | 1. Weight loss-weight maintenance intervention 2. Control group | Body weight measurement before and after diet, 12 months after the end of the diet/GLP-1 levels during standard oral glucose tolerance test before and after the diet and 12 months after the end of the diet /fMRI food-cue reactivity paradigm before and after the diet and 12 months after the end of the diet  ROI: dlPFC | While predicting body weight change over the total observation period, strong interaction between circulating, endogenous GLP-1 levels and dlPFC activity was observed When GLP-1 and dlPFC responses co-occurred, the bodyweight loss was achieved; however, neither GLP-1 nor dlPFC was able to predict body weight loss individually |
| (Farr et al., 2019) | 11 obese men and 9 obese women (BMI ≥ 30 kg/m2) | Double-blind, cross-over, placebo-controlled trial  5 weeks of weekly dose escalation  First phase (either liraglutide or placebo), a minimum 3-week wash-out period, and second phase (received the other option) Each phase included 6 visits/Liraglutide (0.6, 1.2, 1.8. 2,4. 3.0 mg) | 1. Placebo 2. Liraglutide | VAS before and after fMRI scan Food records 3 days per week Stop-Signal Task test Intra-Extra Dimensional Set Shift (IED) test Verbal Recognition Memory (VRM) test  Spatial Span (SSP) test  Spatial Working Memory (SWM) test/Glucose and cholesterol levels/fMRI paradigm fasting, the morning after the last injection | Liraglutide decreased weight, BMI, waist circumference, and fasting glucose, and it improved cholesterol levels  In intra/extra-dimensional set shift, spatial span, spatial working memory or verbal memory neurocognitive testing outcomes, patient's results were not different between liraglutide and placebo group, but liraglutide group showed more success in the stop-signal test  While using liraglutide as compared to placebo, it was observed that OFC showed more activation to food images in comparison to non-food images, when corrected for BMI or weight |
| (Da Porto et al., 2020) | 60 T2DM patients | An open label, prospective controlled study Binge eating disorder (BED) diagnosis Inclusion criteria: Age < 65, 7.5 < HbA1c < 9 Metformin therapy only/Dulaglutide 1.5 mg/sett for 12 weeks | 1. Dulaglutide 2. Gliclazide (60 mg) | Binge eating scale score (BES) Body weight, BMI, percent fat mass/HbA1c levels | Dulaglutide significantly decreased BES scores, body weight, BMI, percent fat mass and HbA1c levels compared to gliclazide Reduced BES scores were directly related with both changes in body weight and HbA1c levels |
| (van Duinkerken et al., 2020) | 10 female candidates for RYGB Age 40-54 years Mean age 47 | Inclusion criteria: BMI > 35 kg/m2 , stable weight during the previous month before surgery (less than 5% reported change) Exclusion criteria: History of neurological disease or psychiatric disorders, using centrally acting agents, having diabetes and MRI contraindications/ Ex9-39 (600 pmol/kg/minute) | 1. Placebo 2. Exendin9-39 | VAS scale for hunger, fullness and prospective food consumption, appetite for sweet, savory or fattening foods/fMRI | Pre-RYGB, Ex-9 resulted in a decrease in connectivity in the right middle frontal gyrus of the right frontoparietal network and an increase in connectivity in the right caudate nucleus of the basal ganglia network Post-RYGB, Ex-9 resulted in a decrease in connectivity in the right OFC of the insula/ACC network Ex-9 more significantly increased the connectivity in the left lateral occipital cortex post-RYGB compared to pre-RYGB and this resulted in a greater decrease in BMI, savory food appetite, and hunger scores |
| **2. Drugs of Abuse** | | | | | |
| (Suchankova et al., 2015) | 908 individuals in the National Institute on Alcohol Abuse and Alcoholism (NIAAA) intramural research program 3802 individuals from Genetics and Environment (SAGE) sample 81 social drinkers 22 alcohol-dependent individuals | Exp1: Alcohol use disorder (AUD) Exp2, 4: Alcohol dependent Exp3:  Intravenous alcohol self-administration (IV-ASA) | Exp1: 1. AUD 2. Control Exp2, 4: 1. Alcohol dependent 2. Nondependent control Exp3:  1. Nondependent | Genotyping analysis Genome-wide association analysis Intravenous alcohol self-administration/ fMRI during monetary incentive delay task Breath alcohol concentration (BrAC) measurements (peak, average, reaching 80 mg%) | 168Ser allele of rs6923761 genotype was associated with AUD in both NIAAA and SAGE sample groups 168Ser/ Ser genotype was associated with higher alcohol intake and BrAC measurements 168Ser/ Ser genotype was associated with increased activity in the right globus pallidus during high monetary reward outcome notification in the incentive delay task |
| (Bouhlal et al., 2017) | 8 healthy humans (age: 43.9 years, BMI: 25.1) | Mean lifetime duration of cocaine use: 21.1 years Age at first cocaine use: 22.2 years Mean cocaine use over 3 months prior to screening: 2–7/week Days used in 14 days prior to screening: 3.9  Participants stayed in the clinic for 13 consecutive days 25 mg IV cocaine was injected via a peripheral venous catheter on day 1, 5, 10 | 1. Cocaine (on day1) 2. Cocaine + oral acetazolamide (on day 5) 3. Cocaine + oral quinine (on day 10) | Subjective response to cocaine (feeling high, rush, elated, stimulated, anxious, wanting cocaine, good drug effect, strong drug effect)/Heart rate, respiratory rate, systolic BP 15 minutes before and after cocaine  Total ghrelin concentration by ELISA Ghrelin, PYY, GLP-1, leptin, insulin, and amylin assay 60 minutes before and after cocaine Plasma cocaine concentration by liquid chromatography-tandem mass spectrometry 15 min after injection Acyl-ghrelin, leptin, insulin, GLP-1, PYY, amylin were by spectrophotometer | GLP-1, PYY levels were significantly reduced after IV cocaine Insulin, amylin levels showed a trend of decrease after IV cocaine No remarkable decrease was detected in acyl-ghrelin, total ghrelin, and leptin concentrations "Anxious" feelings elevated post IV cocaine GLP-1 concentration "High" feelings reduced post IV cocaine PYY concentration Increased respiratory rate decreased post IV cocaine insulin levels Increased heart rate, respiratory rate, and feeling high reduced post IV cocaine amylin levels |
| **3. Reward Learning** | | | | | |
| (Yapici-Eser et al., 2020) | 100 healthy controls 164 schizophrenia (SZ) and bipolar disorder (BPD) patients Ages 18-69 years | Inclusion criteria: Normal intellectual ability, no neurological disorder or heady injury history/7 identified GLP-1R polymorphisms (rs10305420, rs10305421, rs1042044, rs6923761, rs587654, rs761386 and rs10305492) | 1. Healthy control 2. Patients | Response bias and discriminability assessed by probabilistic reward task (PRT) SHAPS, GDA-AA subscales of MASQ, MADRS, PANSS, YMRS self-report scales for anhedonia, anxiety, depression. psychosis, hypomanic symptoms respectively/DNA samples for genome-wide association study (GWAS) analysis | After controlling for age, sex, case-control and discriminability, C allele in rs1042044 was significantly associated with higher response bias in PRT AA genotype of rs1042044 had higher SHAPS scores Other SNPs in GLP-1R were not associated with response bias or SHAPS scores There was no association detected between rs1042044 and depression diagnosis |
